# Design of dual peptide-conjugated hydrogels for proliferation and differentiation of human pluripotent stem cells

**DOI:** 10.1016/j.mtbio.2024.100969

**Published:** 2024-01-23

**Authors:** Tzu-Cheng Sung, Yen-Hung Chen, Ting Wang, Liu Qian, Wen-Hui Chao, Jun Liu, Jiandong Pang, Qing-Dong Ling, Henry Hsin-Chung Lee, Akon Higuchi

**Affiliations:** a State Key Laboratory of Opthalmology, Optometry and Visual Science, Eye Hospital, Wenzhou Medical University, No. 270, Xueyuan Road, Wenzhou, Zhejiang, 325027, China; b Department of Chemical and Materials Engineering, National Central University, No. 300, Jhongda RD., Jhongli, Taoyuan, 32001, Taiwan; c Cathay Medical Research Institute, Cathay General Hospital, No. 32, Ln 160, Jian-Cheng Road, Hsi-Chi City, Taipei, 221, Taiwan; d Department of Surgery, Hsinchu Cathay General Hospital, No. 678, Sec 2, Zhonghua Rd., Hsinchu, 30060, Taiwan; e Graduate Institute of Translational and Interdisciplinary Medicine, National Central University, No. 300, Jhongda Rd., Jhongli, Taoyuan, 32001, Taiwan; f R&D Center for Membrane Technology, Chung Yuan Christian University, Chungli, Taoyuan, 320, Taiwan

**Keywords:** Human pluripotent stem cells, Integrin, Peptide, Hydrogel, Proliferation, Cardiomyocyte

## Abstract

Completely synthetic cell cultivation materials for human pluripotent stem cells (hPSCs) are important for the future clinical use of hPSC-derived cells. Currently, cell culture materials conjugated with extracellular matrix (ECM)-derived peptides are being prepared using only one specific integrin-targeting peptide. We designed dual peptide-conjugated hydrogels, for which each peptide was selected from different ECM sites: the laminin β4 chain and fibronectin or vitronectin, which can target α6β1 and α2β1 or αVβ5. hPSCs cultured on dual peptide-conjugated hydrogels, especially on hydrogels conjugated with peptides obtained from the laminin β4 chain and vitronectin with a low peptide concentration of 200 μg/mL, showed high proliferation ability over the long term and differentiated into cells originating from 3 germ layers *in vivo* as well as a specific lineage of cardiac cells. The design of grafting peptides was also important, for which a joint segment and positive amino acids were added into the designed peptide. Because of the designed peptides on the hydrogels, only 200 μg/mL peptide solution was sufficient for grafting on the hydrogels, and the hydrogels supported hPSC cultures long-term; in contrast, in previous studies, greater than 1000 μg/mL peptide solution was needed for the grafting of peptides on cell culture materials.

## Highlights

Human pluripotent stem cells can proliferate on dual peptide-grafted hydrogels.

## Graphical abstract

**Figure:**
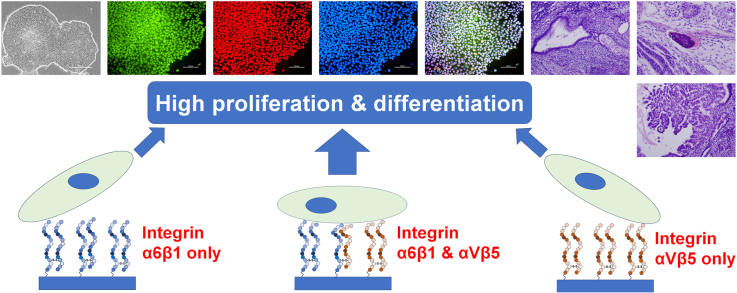

## 1 Introduction

Several clinical trials have incorporated differentiated cells from hPSCs (human pluripotent stem cells), hiPSCs (human induced pluripotent stem cells) or hESCs (human embryonic stem cells) because any types of tissue cells in the human body can be derived from hPSCs with appropriate differentiation protocols [[1], [2], [3], [4], [5], [6], [7]]. For the maintenance and expansion of clinical-grade hPSCs, the cells should be cultured under xeno-free conditions. Unlike conventional cells such as cancer cells or primary cells extracted from tissues, hPSCs cannot, in general, proliferate on conventional dishes made of polystyrene. hPSCs are extensively cultured on extracellular matrix (ECM) proteins, for example, in recombinant vitronectin (rVN)-coated or laminin 521 (LN521)-coated dishes [[8], [9], [10], [11], [12]]. Recently, peptide-conjugated materials have been prepared for hPSC proliferation by several researchers [8,[13], [14], [15], [16], [17], [18], [19], [20], [21], [22], [23], [24], [25], [26], [27], [28]]. Peptide-conjugated cell culture bags, dishes or hydrogels, which can be made in completely xeno-free conditions, are promising biomaterials for hPSC culture and differentiation. The peptides, which are conjugated or coated on cell culture bags or dishes, are designed from cell binding sites of ECM proteins. There are some review articles, which were well summarized peptide-grafted surface that were used for cell culture including hPSCs [25,29].

The peptides are typically derived from vitronectin or laminin for most peptide-conjugated surfaces for hPSC culture [8,[13], [14], [15], [16], [17], [18], [19], [20], [21], [22], [23], [24], [25], [26], [27], [28]]. hPSCs can bind to vitronectin or laminin via integrin receptors of hPSCs, i.e., αVβ5 or α6β1, respectively, and the binding of hPSCs via integrin αVβ5 or α6β1 can maintain the pluripotency of hPSCs. However, most researchers have designed cell culture biomaterials for hPSCs by selecting a single integrin receptor, and they have not yet designed dual peptide-conjugated cell culture materials targeting integrins αVβ5 and α6β1.

Melkoumian et al. conjugated several single peptide-conjugated acrylate surfaces [13]. Among several single peptides, vitronectin-derived peptides, e.g., KGGPQVTRGDVFTMP or PQVTRGDVFTMP, are the most effective for the cultivation of hPSCs on peptide-conjugated acrylate surfaces [[13], [14], [15], [16], [17], [18], [19], [20], [21], [22]]. From their reports [13], they did not insert a joint segment between the material surface and peptide; in contrast, most researchers have reported that the joint segment between the materials or main polymer and the peptide is needed for effective binding between hPSCs and peptide [[23], [24], [25]]. The concentration of the peptide to prepare the peptide-conjugated acrylate surface in Melkoumian's work was 1 mM (1589 μg/mL) in typical experiments [13]. However, compared with that on the surface prepared with 1 mM peptide, the proliferation of hPSCs was 40 % or 20 % peptide-conjugated acrylate surfaces prepared with peptide concentrations of 0.125 mM (199 μg/mL) or 0.063 mM (63 μg/mL) [13]. These concentrations (63–199 μg/mL) are 1–3 orders of magnitude higher than the concentration of ECM such as rVN (5 μg/mL) or LN-521 (10 μg/mL), which were used for coating tissue culture dish surfaces for hPSC culture.

Jia et al. prepared peptide-conjugated polyethylene glycol (PEG) hydrogels [23,24], for which a novel peptide sequence was developed from the investigation of evolutionarily conserved sequences in laminin; however, these researchers investigated the attachment of endothelial cells [23] or hPSC-derived cardiomyocytes [24], not hPSCs, on peptide-conjugated hydrogels. They used 15 mM peptide solution to prepare single peptide-conjugated hydrogels containing GGGGTFALRGDNP at 18,264 μg/mL [23] and GGGGPMQKMRGDVFSP at 24,313 μg/mL [24].

In our previous studies [8,19,[26], [27], [28]], we prepared single peptide-conjugated hydrogels using 1000 μg/mL peptide solution for hPSC proliferation and differentiation. Other investigators [17,19] who prepared peptide-conjugated biomaterials also used a relatively high concentration of peptide (>1000 μg/mL) and only a single peptide-conjugated surface or single ECM-derived peptides-conjugated surface.

In this investigation, we aimed to graft dual peptides on hydrogels for hPSC culture and differentiation that can bind to different types of hPSC integrins, αVβ5 and α6β1 or αVβ5 and α2β1, and evaluated long-term (ten passages) hPSC culture on dual peptide-conjugated hydrogels with the use of minimum concentrations of peptides (less than 1000 μg/mL) for peptide grafting. hPSCs cultured on the dual peptide-conjugated surface created with a minimum concentration of peptides (200 μg/mL) proliferated for more than ten passages and differentiated into not only the cells developed from 3 germ layers but also into a specific cell lineage (cardiac cells), where hPSC-derived cardiac cells are known as weak adhesive cells [24].

## 2 Methods

The animal experiments were performed with the permission of the ethics committees of National Central University (NCU-108-023 and NCU-109-010). Each study was conducted in accordance with all relevant or applicable institutional as well as governmental regulations or guidelines.

### 2.1 Materials

HPS0077 cells (hiPSCs, female) were imported from Riken BioResource Center (Tsukuba, Japan). hESCs (H9) were obtained from WiCell Research Institute (Madison, WI, USA). Supplementary Table 1 lists the biomolecules and materials used in this research. The other biomaterials utilized in the research were supplied from Sigma‒Aldrich (St. Louis, MO, USA).

### 2.2 Development of peptide-conjugated hydrogels

**Fig. 1:**
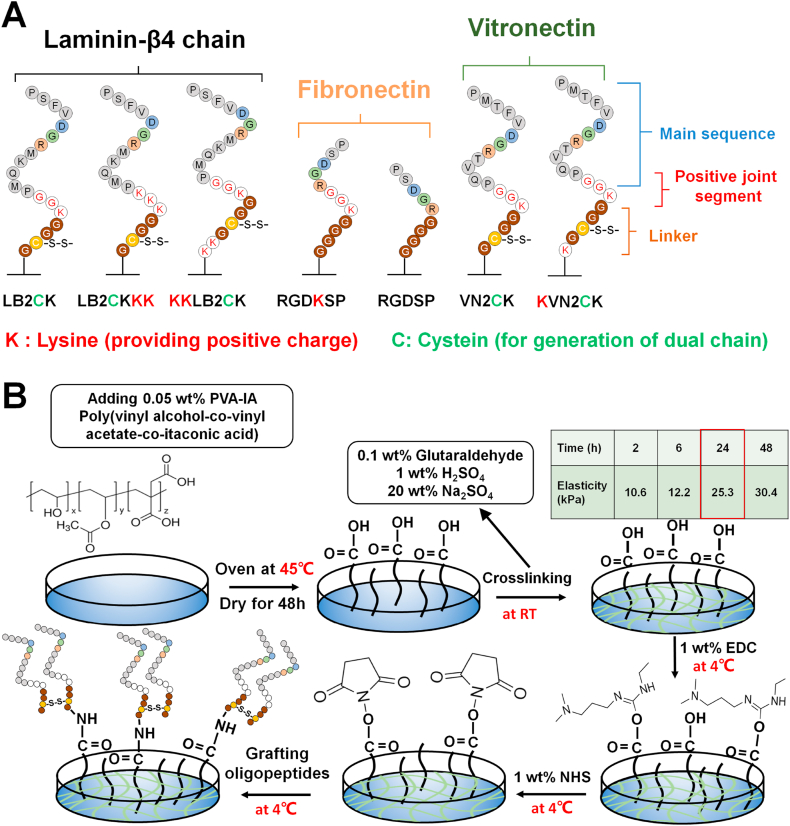
Design of peptide-conjugated hydrogels. (A) Peptide sequences used for the grafting on PVA hydrogels. (B) Preparation method for peptide-conjugated PVA hydrogels. After PVA film was prepared in dishes, PVA was crosslinked with glutaraldehyde. The elasticity of PVA hydrogels was controlled by the reaction time. After the activation of carbonic acid on PVA hydrogels with EDC/NHS, peptide solution was added to the PVA hydrogels for the grafting of the peptides.

We designed several types of peptides, which were conjugated onto poly(vinyl alcohol-*co*-itaconic acid) (PVA; 98 mol% hydrolyzed with 1.3 mol% itaconic acid) hydrogels. The designed peptides were obtained from cell binding sites of laminin β4 (LB2CK, LB2CKKK, and KKLB2CK), fibronectin (RGDKSP and RGDSP) and vitronectin (VN2CK and KVN2CK); the peptide sequences selected for this study are shown in Fig. 1A. Fig. 1B shows preparation method for the peptide-conjugated PVA hydrogels. In this figure, each peptide is shown to have a dual chain triggered by cysteine-cysteine bonding. However, not all of peptides are expected to have a dual chain but have a single chain in several peptides conjugated on PVA hydrogels. The peptides have 3 specific domains: linker (GGGG, GCGGG, KGCGG, or KKGCGG), positive joint segment (KGGG) and main ECM sequence (PMQKMRGDVFSP, RGDSP, or PQVTRGDVFTMP). PVA films were prepared in 6-well tissue culture polystyrene (TCPS) dishes using previously reported methods [[26], [27], [28]]. In brief, 0.05 wt% PVA solution was added to each well of 6-well TCPS dishes, and the dishes were dried to prepare transparent PVA films. The PVA hydrogels were generated by crosslinking the PVA films in an aqueous reaction solution composed of Na_2_SO_4_ (20 w/v%), H_2_SO_4_ (1 w/v%) and glutaraldehyde (0.1 w/v%). The crosslinking intensity of the PVA hydrogels was adjusted by the crosslinking reaction time, which from previous works was determined to be 24 h for the best elasticity for hPSC proliferation and induction [[26], [27], [28]].

PVA hydrogels were conjugated with a single peptide (LB2CK, LB2CKKK, KKLB2CK, RGDKSP, RGDSP, VN2CK, or KVN2CK) or dual peptide (RGDKSP + KKLB2CK or VN2CK + KKLB2CK) using a crosslinking solution of EDC (1-ethyl-3-(-3-dimethylaminopropyl) carbodiimide hydrochloride) and NHS (*N*-hydroxysuccinimide) [[26], [27], [28]]. The PVA hydrogels were activated by a crosslinking solution composed of 10 mg/mL EDC and 10 mg/mL NHS for 6 h at 4 °C, followed by immersion in 20, 50, 100, 200, 500 or 1000 μg/mL aqueous single or dual solution to generate grafting of the peptides onto the PVA hydrogels. The term P–*Y* hydrogels indicates PVA hydrogels conjugated with the *Y* peptide, where *Y* is KKLB2CK, LB2CKKK, LB2CK, RGDKSP, RGDSP, VN2CK, or KVN2CK. For example, P-KKLB2CK hydrogels denote PVA hydrogels conjugated with the KKLB2CK peptide. P-RGDK + KKLB hydrogels indicate PVA hydrogels conjugated with dual peptides of RGDKSP and KKLB2CK. P-VN2C + KKLB hydrogels indicate PVA hydrogels conjugated with dual peptides of VN2CK and KKLB2CK.

### 2.3 Characterization of PVA hydrogel surfaces conjugated with single or mixed designed peptides

The roughness on the PVA hydrogel surface conjugated with single or dual peptides was evaluated with a Primos-CR45 (Camfield Scientific, Parsippany, NJ, USA).

The chemical analysis of the PVA hydrogel surface conjugated with single or dual peptides was performed by X-ray photoelectron spectroscope (XPS, Sigma Probe, Thermo VG-Scientific, UK), where a standard peak maximum of the C 1s spectra was assigned as a binding energy (BE) of 284.6 eV [30,31].

The zeta potential (streaming potential) of the PVA hydrogel surface conjugated with single or mixed designed peptides (20 mm × 10 mm) was evaluated at pH = 7.0 by a SurPASS 3 (Anton Paar, Graz, Austria) electrokinetic analyzer.

### 2.4 hPSC proliferation on PVA hydrogels conjugated with single or mixed designed peptides under xeno-free proliferation protocols

HPS0077 cells (hiPSCs) or H9 (hESCs) were expanded on a recombinant vitronectin (rVN) (5 μg/mL)-coated dish surface in xeno-free Essential 8 (E8) medium (xeno-free medium) utilizing a conventional protocol [[32], [33], [34], [35]]. The cells were seeded on PVA hydrogels conjugated with single or mixed designed peptides in E8 medium at a density of 5 × 10^4^ cells per cm^2^, and the medium was replaced with fresh medium every day during cell proliferation. After hPSC proliferation for approximately one week until approximately 80–90 % confluent, hPSCs were passaged, utilizing conventional protocols as described below, onto a new PVA hydrogel surface conjugated with designed single or dual peptides.

hPSCs on PVA hydrogels were treated with dispase II solution (2 mg/mL) for 5–10 min in a CO_2_ incubator at 25 °C to make the cells detached from the PVA hydrogels. Then, we could observe the edge of the colonies were peeling off and could be detached by pipetting with DMEM/F12 solution. The cells were collected in a conical tube and then centrifuged with 160×*g* for 5 min at room temperature. Subsequently, the solution phase in the conical tube was replaced by Essential 8 medium and pipetted gently. Simultaneously, the cells became into small colony pieces and could be seeded on a new peptide-conjugated PVA hydrogels.

### 2.5 hPSC expansion and pluripotency

Fold expansion of hPSCs on a PVA hydrogel surface conjugated with single or mixed designed peptides was calculated using the following equation [[32], [33], [34], [35]]:

Fold expansion = *N*(after)/*N*(initial). (1)

where *N*(after) indicates the number of hPSCs on the hydrogel surface after proliferation and *N*(initial) is the initial number of hPSCs seeded on the hydrogel surface. It should be noted that Fold expansion (*X*) is directly related to doubling time (*T*) after *Z* days cultivation of hPSCs by the following equation:

(2) *T* = ln(2)*Z/ln(*X*)

Differentiated cells were analyzed with flow cytometry to determine whether the cells expressed the pluripotent marker SSEA-4. The differentiation rate of hPSCs after proliferation on the PVA hydrogel surface conjugated with designed single or dual peptides was evaluated for each passage using the following equation:

(3) Differentiation rate (%) = 100% - *P*(SSEA-4) (%)

where *P*(SSEA-4) is the SSEA-4 expression in hPSCs after hPSC culture on the PVA hydrogel surface conjugated with designed single or dual peptides.

Pluripotent protein (Oct3/4, Nanog, Sox-2 and SSEA-4) expression in hPSCs was examined by immunohistochemical labelling, following a conventional procedure, using specific antibodies for the pluripotent proteins [8,28,32,33,[35], [36], [37], [38]]. The labelled cells were assessed with a fluorescence microscope (Eclipse Ti–U, Nikon Instruments, Tokyo, Japan).

### 2.6 Teratoma assay

Teratoma formation was assessed following a conventional protocol [[32], [33], [34], [35],^39^,^40^] after hPSCs proliferated on a PVA hydrogel surface conjugated with designed single or dual peptides for 10 passages. Briefly, hPSCs were detached from the peptide-conjugated PVA hydrogels and centrifuged to make cell pellets. The cell pellets were subsequently inserted into DMEM/F12 medium with Matrigel. Nonobese diabetic/severe combined immunodeficiency (NOD.CB17-Prkdcscid/JNarl NOD/SCID) mice were subcutaneously transplanted with a total of 5.0 × 10^6^ hPSCs. After 6–9 weeks, the teratomas were typically observed, then taken out and fixed in a formaldehyde solution. The paraffin-embedded teratoma was sliced, and the sliced teratomas were stained with hematoxylin and eosin (H & E) following a conventional method [[32], [33], [34], [35],^39^,^40^].

### 2.7 Differentiation of hiPSCs into cardiomyocytes

After proliferation on PVA hydrogels conjugated with designed single or dual peptides under xeno-free proliferation conditions for ten passages, hiPSCs were induced to differentiate into cardiomyocytes following a method published by Sharma et al. [41] with some revisions. Detailed induction method into cardiomyocytes was described in our previous work [42].

### 2.8 Characterization of hiPSC-derived cardiomyocytes

The expression of troponin T, a cardiac marker (cTnT), on cardiomyocytes obtained from hiPSCs was analyzed utilizing flow cytometry following a conventional protocol [43] that included a primary antibody solution (1:200 dilution) containing cTnT or an isotype antibody solution (IgG1 isotype-mouse antibody) and a secondary antibody solution (1:1000 dilution) containing Alexa Fluor 488 goat anti-mouse IgG [44].

Immunohistochemical staining of cardiomyocytes derived from hiPSCs was performed for cardiac marker proteins (cTnT) using a conventional protocol, for which primary antibodies against cTnT (MA5-12960; mouse, 1:200 dilution, green) and secondary antibodies against Alexa Fluor 488 goat anti-mouse IgG antibody (A11001; 1:500 dilution) were used [44]. The expression of cTnT as well as the nuclei (stained with DAPI) were evaluated with confocal laser microscope (A1R HD, Nikon Instruments, Tokyo, Japan) [44].

### 2.9 Statistical analysis

For each experiment, the results from four samples were analyzed. The results are shown as means ± standard deviations. Statistical evaluations were performed utilizing one-way ANOVA with a post hoc *t*-test. The Tukey‒Kramer post hoc test was also performed after one-way ANOVA. Probability (*p*) values less than 0.05 were considered statistically significant.

## 3 Results and discussion

### 3.1 Design of peptides conjugated on PVA hydrogels

We designed peptides that originated from the laminin β4 chain (LB2CK, LB2CKKK, and KKLB2CK), fibronectin (RGDKSP and RGDSP), and vitronectin (VN2CK and KVN2CK) (Fig. 1A). Most researchers have used peptides derived from vitronectin (PQVTRGDVFTMP or KGGPQVTRGDVFTMP) for the preparation of peptide-conjugated surfaces [[13], [14], [15], [16], [17], [18], [19], [20], [21], [22]], where hPSCs can proliferate. The peptide derived from fibronectin (RGDSP) is typically used to enhance the adhesion of any type of cell [[45], [46], [47], [48], [49], [50], [51], [52], [53], [54]] but cannot effectively support hPSC proliferation on RGDSP-conjugated surfaces. The peptide derived from the laminin β4 chain (PMQJMRGDVFSP) was originally developed by Jia et al. for the adhesion of hPSC-induced cardiomyocytes [24]; this peptide was effective for hPSC-derived cardiomyocyte adhesion (but not pluripotent hPSC adhesion) and was shown to be useful for hPSC proliferation on PMQJMRGDVFSP-conjugated surfaces in our previous studies [8,28]. However, previous studies used a high concentration (1000 μg/mL) of peptides for grafting onto PVA hydrogels [8,28]. Our designed peptides have a GGGG or GCGG linker on each main sequence (Fig. 1A). Typically, the peptide sequence of four glycines (GGGG) is a preferable length of linker between the cell adhesion peptide and the material surface or main polymer chain [24] because of the flexibility of the GGGG chain. Cysteine in the GCGG chain is expected to contribute to the generation of a dual peptide chain on the peptide-conjugated surface because of S–S binding. We also added a positive joint segment, K, KK, KGG, and KKK, to most of the peptides in this study (Fig. 1A); this segment is expected to enhance the zeta potential of cell culture biomaterials.

We conjugated several peptides onto PVA hydrogels using EDC/NHS chemistry utilizing a single peptide solution or dual peptide solution (RGDKSP + KKLB2CK or VN2CK + KKLB2CK), where P-RGDK + KKLB hydrogels were prepared by grafting RGDKSP and KKLB2CK using the same concentration of each peptide. P-VN2C + KKLB hydrogels were prepared by grafting VN2CK and KKLB2CK using the same concentration of each peptide.

### 3.2 Characteristic properties of PVA hydrogels conjugated with designed peptides

The chemical analysis of the surface of peptide-conjugated PVA hydrogels was performed using XPS. This method can be used to indirectly demonstrate the existence of peptide on the PVA hydrogel surfaces because PVA hydrogels do not contain nitrogen or sulfur atoms but are composed solely of hydrogen, oxygen and carbon, with the peptides containing nitrogen and/or sulfur atoms. The high-resolution XPS spectra of the C 1s (Supplementary Fig. 1A), N 1s (Supplementary Fig. 1B), and S 2p (Supplementary Fig. 1C) peaks on the surfaces of the peptide-conjugated PVA hydrogels were examined; the spectra are shown in Supplementary Fig. 1. The C 1s peak of the peptide-conjugated PVA hydrogels was wider than the C 1s peak of the non-peptide-conjugated PVA hydrogels. N 1s and S 2p peaks of the peptide-conjugated PVA hydrogels were present extensively, whereas no distinct N 1s or S 2p peaks were observed for unconjugated PVA hydrogels (Supplementary Fig. 1). This finding can be explained by the fact that the peptide-conjugated PVA hydrogels contain nitrogen and sulfur atoms derived from peptides whereas nonconjugated PVA hydrogels contain solely carbon, oxygen and hydrogen atoms and do not include nitrogen or sulfur atoms. These observations support the idea that the peptides were conjugated onto the peptide-conjugated PVA hydrogel surface.

**Fig. 2:**
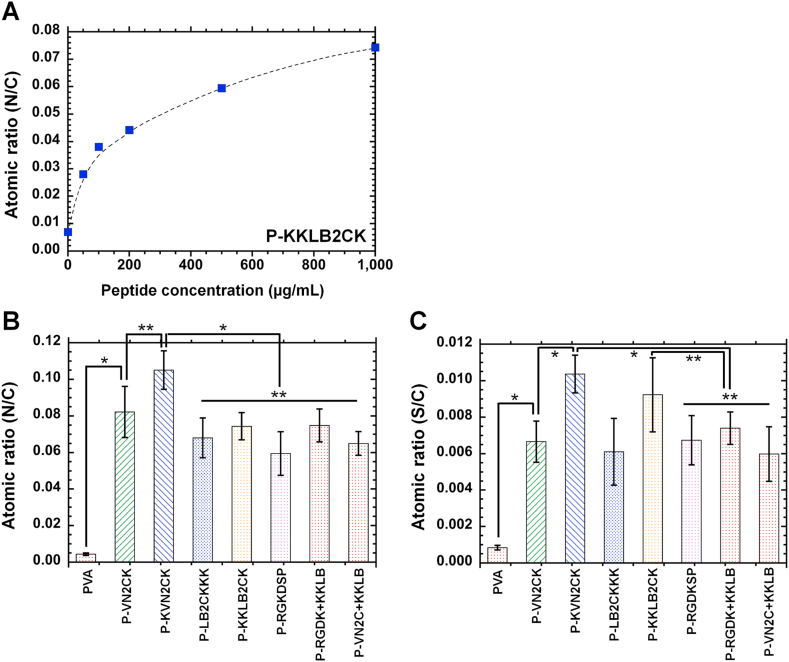
XPS analysis of peptide-conjugated PVA hydrogels. (A) Nitrogen to carbon (N/C) atomic ratio for the surface of P-KKLB2CK hydrogels prepared with several concentrations of KKLB2CK peptide. (B) Nitrogen to carbon (N/C) atomic ratios for the surfaces of PVA (PV) hydrogels and peptide-conjugated PVA hydrogels. *p < 0.05. **p > 0.05. (C) Sulfur to carbon (S/C) atomic ratios for the surfaces of PVA (PV) hydrogels and peptide-conjugated PVA hydrogels. *p < 0.05. **p > 0.05.

The N/C atomic ratio on the P-KKLB2CK surface, which was prepared with various peptide concentrations (0–1000 μg/mL) of KKLB2CK, was investigated; the results are displayed in Fig. 2A. The N/C ratio increased with increasing concentrations of KKLB2CK, suggesting that the surface density of KKLB2CK increased with the peptide concentration of KKLB2CK during the peptide grafting reaction on PVA hydrogels. Fig. 2B and C shows the atomic ratios of N/C and S/C, respectively, on the surfaces of the peptide-conjugated PVA hydrogels, which were prepared with 1000 μg/mL of each peptide. The N/C ratio on the surfaces of the peptide-conjugated PVA hydrogels ranged from 0.06 to 0.105, which was much higher than that (approximately 0.01) on the nonconjugated PVA hydrogels (*p* < 0.05). The trend for the S/C ratio on the surfaces of the peptide-conjugated PVA hydrogels was similar to the trend for the N/C ratio on the surface of the peptide-conjugated PVA hydrogels; S/C ratio on the surfaces of the peptide-conjugated PVA hydrogels ranged from 0.006 to 0.0105, which was much higher than that (approximately 0.001) on the nonconjugated PVA hydrogels (*p* < 0.05). In most cases, we observed no extensive difference in the N/C ratio or S/C ratio among the peptide-conjugated PVA hydrogels. In particular, there were no extensive differences in the N/C ratio or S/C ratio on the surfaces of dual peptide-conjugated PVA hydrogels and single peptide-conjugated PVA hydrogels (*p* > 0.05).

These results can be explained by both dual peptide-conjugated PVA hydrogels and single peptide-conjugated PVA hydrogels being prepared with the same NHS/EDC reactions utilizing the same peptide concentrations in the reaction solution as well as the same reaction time for the grafting of the peptides on the PVA hydrogels. Because of the higher N/C and S/C ratios on the surfaces of peptide-conjugated PVA hydrogels than on the surfaces of the nonpeptide-conjugated PVA hydrogels, we can conclude that single or dual peptides were conjugated onto the surface of PVA hydrogels.

The surface roughness of the peptide-conjugated PVA hydrogels was studied using a surface roughness instrument (Primos-CR45); the results are shown in Supplementary Fig. 2A. We found no significant difference in surface roughness between the nonconjugated PVA hydrogels and peptide-conjugated PVA hydrogels. The peptide-conjugated PVA hydrogels had relatively smooth surfaces. The root mean square (RMS) roughness for each hydrogel surface was studied; the results are displayed in Supplementary Fig. 2B. The RMS roughness of the peptide-conjugated PVA hydrogels was estimated to be approximately 10–25 μm, comparable to the roughness of the tissue culture polystyrene dish surface reported in the literature (24 μm) [8].

**Fig. 3:**
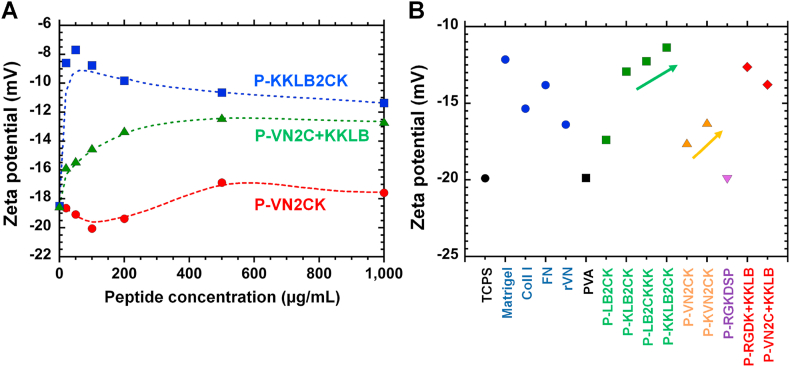
Physical characterization of peptide-conjugated PVA hydrogels. (A) Zeta potential of the peptide-conjugated PVA (P-KKLB2CK, PVN2CK, and P-VN2C + KKLB) hydrogels prepared with several peptide concentrations. (B) Zeta potentials of peptide-conjugated PVA hydrogel surfaces and ECM-coated dishes.

The zeta potential and surface electrical potential of the peptide-conjugated PVA hydrogels were also analyzed (Fig. 3A and B). A material surface with high negative potential is considered not preferable for cell adhesion and proliferation because hPSCs have a negative electrical potential (approximately −70 mV) under resting conditions. However, a surface with an extensively high positive electrical potential could induce nonspecific hPSC adhesion on the surfaces, leading to difficulty maintaining the pluripotent state of hPSCs. Therefore, an optimal electrical potential (zeta potential) of material surfaces for hPSC adhesion and for the maintenance of pluripotent conditions is important for cell cultivation materials.

The effect of the zeta potential on the surfaces of single peptide-conjugated PVA hydrogels (P-KKLB2CK and P-VN2CK) and dual peptide-conjugated PVA hydrogels (P-VN2C + KKLB) on the peptide concentration for grafting on PVA hydrogels was investigated; the results are shown in Fig. 3A. Because KKLB2CK has three additional positive amino acids (lysine) and VN2CK has only one additional positive amino acid (lysine), the zeta potential of P-KKLB2CK was 7–10 mV higher than that of P-VN2CK. P-KKLB2CK hydrogels prepared with a low concentration of KKLB2CK solution, such as 20 μg/mL, showed a relatively high zeta potential compared to the zeta potential for P-VN2CK. The zeta potential for P-VN2C + KKLB showed in the middle of the zeta potential for P-VN2CK and P-KKLB2CK at the same peptide concentration for the preparation of the peptide-conjugated PVA hydrogels.

The zeta potential of the surfaces of the peptide-conjugated PVA hydrogels, which were prepared with several designed single or dual peptides, ECM protein-coated TCPS dishes, nonmodified TCPS dishes and nonconjugated PVA hydrogels was investigated; the results are shown in Fig. 3B. The nontreated TCPS and PVA hydrogels showed relatively low zeta potentials of approximately −20 mV. The zeta potential of ECM-coated TCPS dishes ranged from −17 mV to −12 mV. The zeta potential of the surface of the peptide-conjugated PVA hydrogels ranged from −17.5 mV to −11 mV, which is much higher than that for nontreated TCPS and nonconjugated PVA hydrogels and comparable to that for ECM-coated TCPS dishes. In general, the zeta potential of peptide-conjugated PVA hydrogels with the same main chain showed a higher zeta potential when the peptides contained more positively charged amino acids (lysine) (Fig. 3B).

### 3.3 hiPSC cultivation on single and dual peptide-conjugated PVA hydrogels

**Fig. 4:**
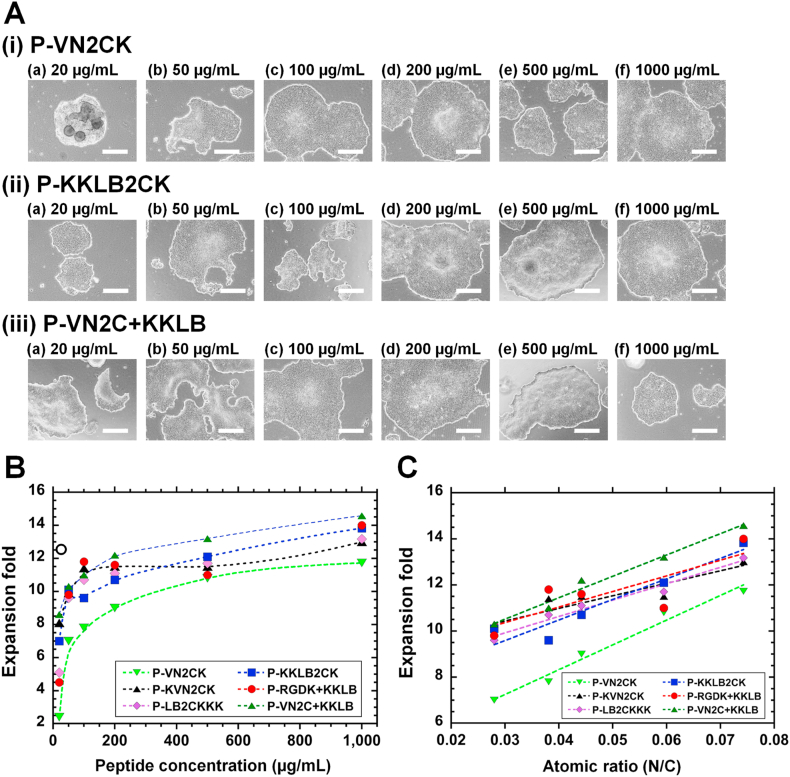
hiPSC (HPS0077) culture on single or dual peptide-conjugated PVA hydrogels cultured under xeno-free proliferation conditions. (A) Morphologies of HPS0077 on P-VN2CK (i), P-KKLB2CK (ii), and P-VN2C + KKLB (iii) hydrogels prepared with peptide concentrations of 20 (a), 50 (b), 100 (c), 200 (d), 500 (e), and 1000 (f) μg/mL at passage 3. The scale bar represents 500 μm. (B) Dependence of fold expansion of HPS0077, cultured on single and dual peptide-conjugated PVA hydrogels, during passages 1–3 on the concentration of peptides used for the grafting of peptides to PVA hydrogels. Fold expansion of hiPSCs on T-rVN dishes is also plotted as open circle. (C) Dependence of fold expansion of HPS0077, cultured on single and dual peptide-conjugated PVA hydrogels, during passage 1–3 on the nitrogen to carbon atomic ratios for peptide-conjugated PVA hydrogels, as analyzed by XPS.

HPS0077 cells (hiPSCs) were cultivated on single peptide-conjugated PVA hydrogels (P-VN2CK, P-KKLB2CK) and dual peptide-conjugated PVA hydrogels (P-VN2C + KKLB) for 3 passages under a variety of peptide concentrations (20, 50, 100, 200, 500 and 1000 μg/mL). The colony size of hiPSCs on peptide-conjugated PVA hydrogels increased with increasing peptide concentration in the grafting solution (Fig. 4A). However, HPS0077 cells on P-VN2C + KKLB hydrogels prepared with 1000 μg/mL at passage 3 were found to show relatively small colonies. This is probably because hiPSCs attached strongly on P-VN2C + KKLB hydrogels, which leads to generate small colonies due to the usage of more strong enzyme digestion time compared to hiPSCs on the peptide-conjugated hydrogels prepared with lower concentration of peptide solution (e.g., 20–500 μg/mL). The fold expansion of hiPSCs on peptide-conjugated PVA hydrogels prepared with grafting solution with varying peptide concentrations was investigated; fold expansion was the average fold expansion for 3 passages (Fig. 4B). The fold expansion of hiPSCs increased with increasing peptide concentration in the grafting solution, a finding that supports the dependence of hiPSC colony size on concentration found in Fig. 4A. Because the atomic ratio of N/C on peptide-conjugated PVA hydrogels increased with increasing peptide concentration in the grafting solution, we evaluated the dependence of the fold expansion of hiPSCs cultivated on peptide-conjugated PVA hydrogels on the atomic ratio of N/C on peptide-conjugated PVA hydrogels, which were prepared with grafting solution with varying peptide concentrations; fold expansion was the average fold expansion for 3 passages (Fig. 4C). The results indicated excellent linear correlations (e.g., the correlation coefficients for P-VN2CK, P-KVN2CK, P-LB2CKKK, P-KKLB2CK, P-RGDK + KKLB, and P-VN2C + KKLB were 0.99, 0.91, 0.98, 0.95, 0.81 and 0.99, respectively). hiPSCs proliferate more on PVA hydrogels conjugated with a higher density of ECM-derived peptides. Dual peptide-conjugated (P-VN2C + KKLB) PVA hydrogels showed the highest fold expansion of hiPSCs among peptide-conjugated PVA hydrogels prepared with the same peptide concentration. However, the dual peptide-conjugated (P-RGDK + KKLB) PVA hydrogels showed a relatively low fold expansion of hiPSCs among peptide-conjugated PVA hydrogels prepared with the same peptide concentration. This occurred because hiPSCs cannot attach to and proliferate on P-RGDKSP hydrogels. Therefore, hiPSCs on P-RGDK + KKLB mainly attached to the KKLB2CK peptide on PVA hydrogels, indicating that the fold expansion on P-RGDK + KKLB hydrogels prepared at a concentration of *X* is expected to be similar to that on P-KKLB2CK hydrogels prepared with a half concentration of peptides (*X*/2).

### 3.4 Long-term proliferation of hiPSCs on single and dual peptide-conjugated PVA hydrogels

**Fig. 5:**
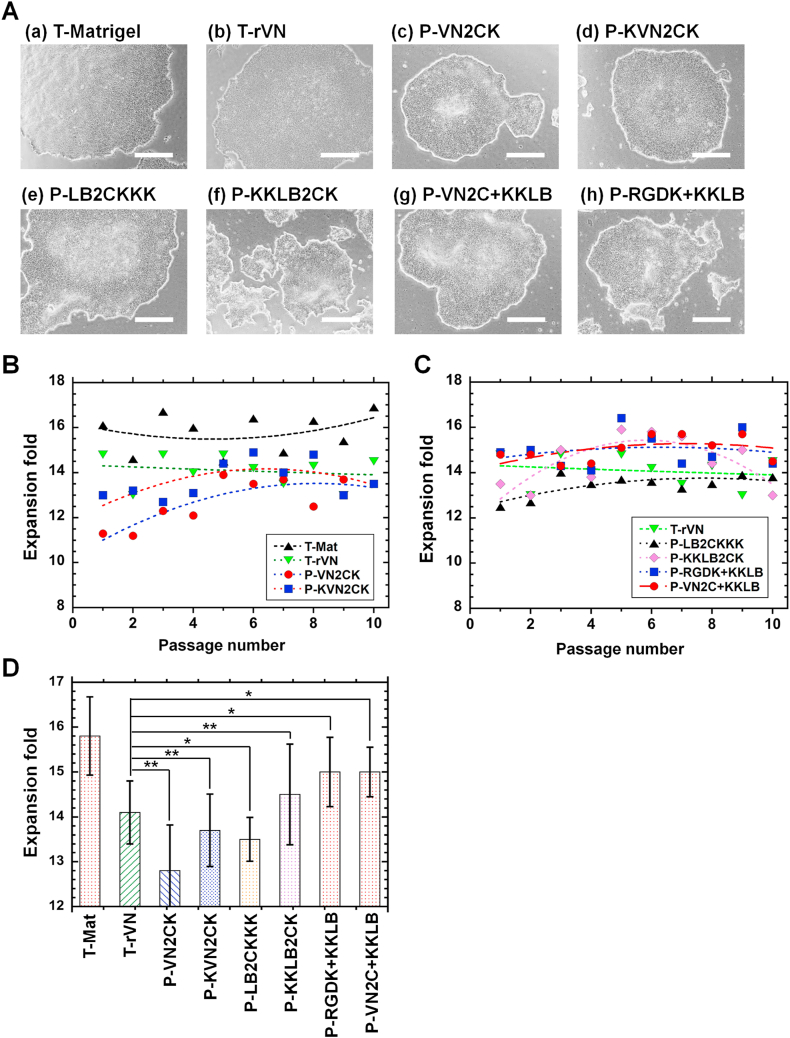
Long-term proliferation of hiPSCs (HPS0077) on peptide-conjugated PVA hydrogels under xeno-free proliferation conditions. (A) Morphologies of hiPSCs on Matrigel-coated dishes (a), rVN-coated dishes (b), P-VN2CK hydrogels (c), P-KVN2CK hydrogels (d), P-LB2CKKK hydrogels (e), P-KKLB2CK (f), P-VN2C + KKLB hydrogels (g), and P-RGDK + KKLB hydrogels (h) at passage ten. The scale bar represents 500 μm. (B) Dependence of fold expansion of hiPSCs on passage on Matrigel-coated dishes (closed black triangle), rVN-coated dishes (closed reverse green triangle), P-VN2CK hydrogels (closed red circle), and P-KVN2CK hydrogels (closed blue square). (C) Dependence of fold expansion of hiPSCs on passage on rVN-coated dishes (closed reverse green triangle), P-LB2CKKK hydrogels (closed black triangle), P-KKLB2CK hydrogels (closed purple rhombus), P-RGDK + KKLB hydrogels (closed blue square), and P-VN2C + KKLB hydrogels (closed red circle). (D) Average fold expansion of hiPSCs on rVN-coated dishes and single or dual peptide-conjugated PVA hydrogels for 10 passages. *p < 0.05. **p > 0.05. (For interpretation of the references to color in this figure legend, the reader is referred to the Web version of this article.)

Long-term hiPSC proliferation experiments were performed on single and dual peptide-conjugated PVA hydrogels, where the peptide concentration in the reaction solution for grafting on PVA hydrogels was 200 μg/mL, which is significantly less than the concentration (>1000 μg/mL) used in previous studies [8,17,19,[26], [27], [28]]. Fig. 5A shows the cell morphologies of hiPSCs on the single and dual peptide-conjugated PVA hydrogels after 10 passages. hiPSCs proliferated on all single or dual peptide-conjugated PVA hydrogels developed in this study. The dependence of passage number on the fold expansion of hiPSCs cultured on single and dual peptide-conjugated PVA hydrogels as well as on Matrigel-coated and rVN-coated dishes (positive control) is shown in Fig. 5B and C. The fold expansion of hiPSCs on Matrigel-coated surfaces was highest during the 10 passages. However, the culture of hiPSCs on Matrigel-coated surfaces is not xeno-free and not a chemically defined condition, which is not a preferable culture condition for hiPSCs for clinical use. Therefore, hiPSC expansion on rVN-coated dishes should serve as the control for the future clinical use of hiPSCs. The average fold expansion of hiPSCs during 10 passages was calculated on T-rVN (rVN-coated dishes, control experiments) and single or dual peptide-conjugated PVA hydrogels (Fig. 5D). The average fold expansion of hiPSCs on dual peptide-conjugated PVA hydrogels, P-RGDK + KKLB hydrogels and P-VN2C + KKLB hydrogels was statistically higher than that on rVN-coated (T-rVN) dishes (*p* < 0.05), which was the control experiments in xeno-free culture conditions. The fold expansion of hiPSCs on any single peptide-conjugated PVA hydrogel was similar or less than that of hiPSCs on rVN-coated dishes.

The higher fold expansion of hiPSCs on dual peptide-conjugated PVA hydrogels (P-RGDK + KKLB and P-VN2C + KKLB hydrogels) than on single peptide-conjugated PVA hydrogels can be explained as follows: hiPSCs can bind to dual peptide-conjugated PVA hydrogels with αVβ5 integrin via the VN2CK peptide and with α6β1 integrin via the KKLB2CK peptide on P-VN2C + KKLB hydrogels; hiPSCs can bind to dual peptide-conjugated PVA hydrogels with fibronectin binding integrin via the RGDKSP peptide and with α6β1 integrin via the KKLB2CK peptide on P-RGDK + KKLB hydrogels. Dual binding sites via different integrins should have a positive effect on the proliferation of hiPSCs on dual peptide-conjugated PVA hydrogels.

hESCs (H9) were also cultivated on single and dual peptide-conjugated PVA hydrogels, where the peptide concentration in the reaction solution for grafting on PVA hydrogels was 200 μg/mL for 10 passages. Supplementary Fig. 3A shows the cell morphologies of hESCs on the single and dual peptide-conjugated PVA hydrogels after 10 passages. hESCs proliferated on all single or dual peptide-conjugated PVA hydrogels developed in this study. The dependence of passage number on the fold expansion of hESCs cultured on single and dual peptide-conjugated PVA hydrogels as well as on rVN-coated dishes (positive control) is shown in Supplementary Figs. 3B and 3C hESCs on P-VN2C + KKLB hydrogels proliferate better than those on rVN-coated dishes.

The average fold expansion of hESCs during 10 passages was calculated on T-rVN (rVN-coated dishes, control experiments) and single or dual peptide-conjugated PVA hydrogels (Supplementary Fig. 3D). The average fold expansion of hiPSCs on dual peptide-conjugated PVA hydrogels, P-VN2C + KKLB hydrogels was statistically higher than that on rVN-coated (T-rVN) dishes (*p* < 0.05), which was the control experiments in xeno-free culture conditions.

### 3.5 Characterization of hiPSCs after long-term proliferation on single and dual peptide-conjugated PVA hydrogels

It is necessary to study whether hiPSCs can sustain their pluripotent characteristics and retain the ability to induce into cells generated from 3 germ layers after long-term proliferation on single and dual peptide-conjugated PVA hydrogels, which were developed in this study.

**Fig. 6:**
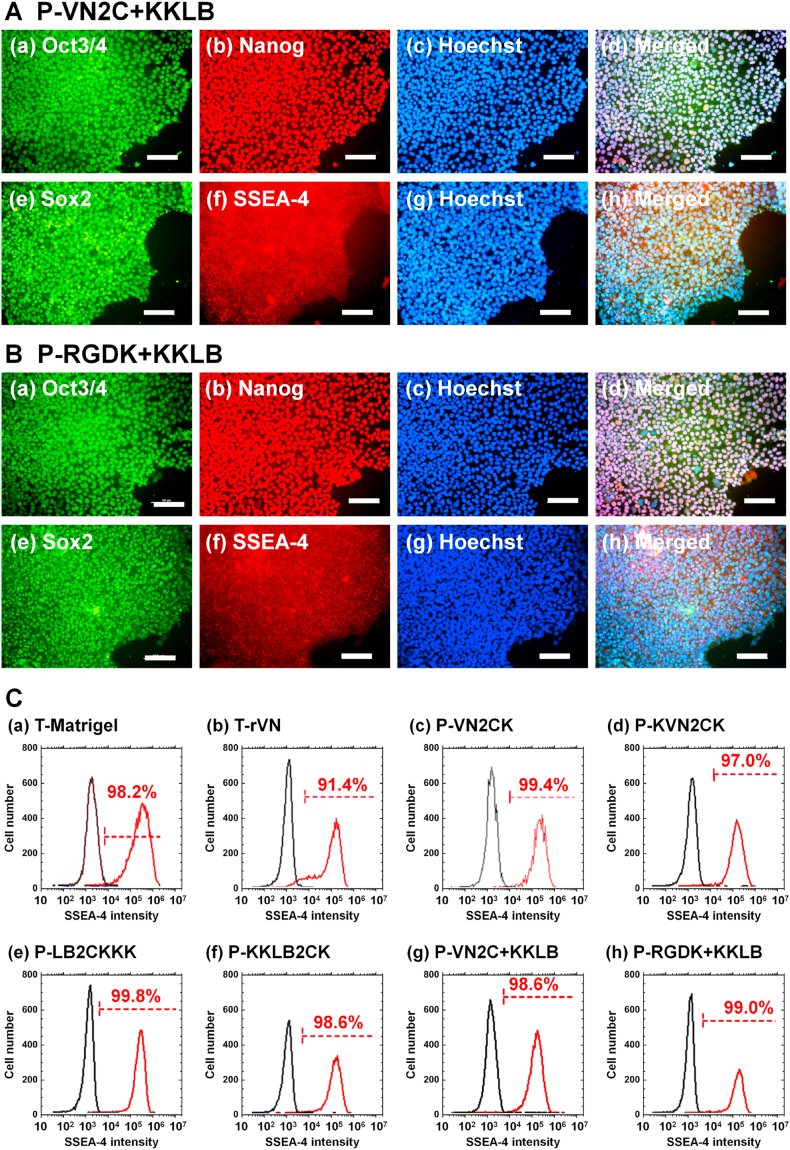
Analysis of the pluripotency of hiPSCs (HPS0077) after long-term (ten passages) proliferation on single or dual peptide-conjugated PVA hydrogels under xeno-free proliferation conditions. (A, B) Expression of the pluripotent proteins Oct3/4 (a, green), Nanog (b, red), Sox2 (e, green), and SSEA-4 (f, red) in hiPSCs, as determine with immunostaining and nuclear staining (Hoechst 33342) (blue, c, g) after the long-term (ten passages) proliferation of hiPSCs on P-VN2C + KKLB (A) and P-RGDK + KKLB (B) hydrogels. The images in (d) and (h) were generated by merging (a)–(c) and (e)–(g), respectively. The scale bar represents 100 μm. (C) Flow cytometry analysis of pluripotent marker (SSEA-4) expression in hiPSCs after long-term (ten passages) proliferation on Matrigel-coated dishes (a), rVN-coated dishes (b), P-VN2CK hydrogels (c), P-KVN2CK hydrogels (d), P-LB2CKKK hydrogels (e), P-KKLB2CK (f), P-VN2C + KKLB hydrogels (g), and P-RGDK + KKLB hydrogels (h). (For interpretation of the references to color in this figure legend, the reader is referred to the Web version of this article.)

The expression of pluripotent proteins (Oct3/4, SSEA-4, Nanog, and Sox2) on hiPSCs after proliferation on single and dual peptide-conjugated PVA hydrogels (P-RGDK + KKLB and P-VN2C + KKLB) for ten passages under xeno-free conditions was studied utilizing an immunohistochemical staining method; the results are displayed in Fig. 6A for hiPSCs on P-VN2C + KKLB hydrogels, Fig. 6B for hiPSCs on P-RGDK + KKLB hydrogels, Supplementary Fig. 4A for hiPSCs on P-LB2CKKK hydrogels, Supplementary Fig. 4B for hiPSCs on P-KKLB2CK hydrogels, Supplementary Fig. 5A for hiPSCs on P-VN2CK hydrogels, and Supplementary Fig. 5B for hiPSCs on P-VN2CK hydrogels. Hoechst 33342 was used to stain hiPSC nuclei. hiPSCs showed extensive pluripotent protein expression even after long-term proliferation (ten passages) on single and dual peptide-conjugated PVA hydrogels (P-LB2CKKK, P-KKLB2CK, P-VN2CK, P-KVN2CK, P-RGDK + KKLB and P-VN2C + KKLB hydrogels).

The pluripotency of hiPSCs can be quantitatively evaluated by the expression of pluripotent markers using flow cytometry. Therefore, the expression of the pluripotent marker SSEA-4 in hiPSCs after 10 passages on several single or dual peptide-conjugated PVA hydrogels was evaluated operating flow cytometry; the results are depicted in Fig. 6C hiPSCs proliferated on both single- and dual peptide-conjugated PVA hydrogels, and more than 97 % exhibited SSEA-4 expression, indicating that hiPSCs can sustain their pluripotency after long-term proliferation on both single and dual peptide-conjugated PVA hydrogels. We also evaluated Oct3/4 and Sox2 expression of hiPSCs after 10 passages on dual peptide-conjugated PVA hydrogels (P-VN2C + KKLB hydrogels) and on rVN-coated dishes. The results are shown in Supplementary Fig. 6 hiPSCs proliferated on dual peptide-conjugated PVA hydrogels (P-VN2C + KKLB hydrogels) and rVN-coated dishes showed more than 95 % and 91 % expression of Oct3/4 and Sox2, respectively, indicating that hiPSCs can sustain their pluripotency after long-term proliferation on dual peptide-conjugated PVA hydrogels as well as on rVN-coated dishes.

**Fig. 7:**
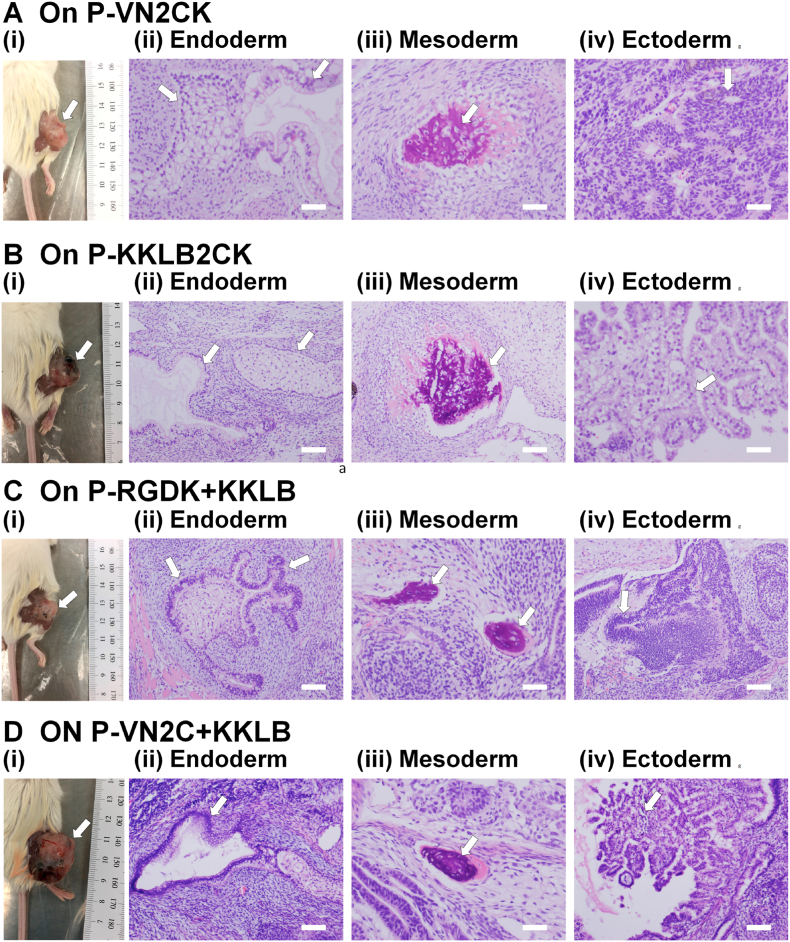
Differentiation ability of hiPSCs (HPS0077) *in vivo* utilizing a teratoma analysis after long-term (ten passages) proliferation on single or dual peptide-conjugated PVA hydrogels under xeno-free proliferation conditions. (A–D) (i) A teratoma created by the transplantation of HPS0077. (ii-iv) Glandular ducts and squamous nests (ii, endoderm), bone-like tissue (iii, mesoderm), and immature neuroepithelium and choroid plexus-like tissue (iv, ectoderm) were detected. The white arrows indicate a teratoma (i) and specific tissues (ii, iii and iv) on P-VN2CK (A), P-KKLB2CK (B), P-RGDK + KKLB (C), and P-VN2C + KKLB (D) hydrogels. The scale bar represents 100 μm (B(ii), B(iii), C(i), C(iii), D(i), D(iii)) or 200 μm (A(ii)-A(iv), B(iv), C(iii), D(iii)).

The differentiation potential of hiPSCs into cells originating from 3 germ layers *in vivo* is also valuable for evaluating whether hiPSCs can sustain their pluripotency after long-term proliferation on single or dual peptide-conjugated PVA hydrogels. Therefore, teratoma formation in NOD/SCID (NOD. CB17-*Prkdc*^scid^/Jnarl) mice was generated by hiPSC injection to study the potential of these cells to induce into cells originating from 3 germ layers (*in vivo* assay); the hiPSCs were injected after 10 passages on P-VN2CK (Fig. 7A), P-KKLB2CK (Fig. 7B), P-RGDK + KKLB (Fig. 7C) and P-VN2C + KKLB (Fig. 7D) hydrogels. We observed endoderm-derived cells (glandular duct; Fig. 7A(ii), 7B(ii), 7C(ii), and 7D(ii), squamous nest; Fig. 7A(ii) and 7C(ii)), mesoderm-derived cells (bone-like tissue; Fig. 7A(iii), 7B(iii), 7C(iii), and 7D(iii)), and ectoderm-derived cells (immature neuroepithelium; Fig. 7A(iv) and 7C(iv), choroid plexus-like tissue; Fig. 7B(iv) and 7D(iv)). Therefore, hiPSCs differentiated into 3 germ layers *in vivo*, even after proliferation on single and dual peptide-conjugated PVA hydrogels for 10 passages under xeno-free proliferation conditions.

### 3.6 Cardiac cell differentiation of hiPSCs after long-term proliferation on single and dual peptide-conjugated PVA hydrogels

**Fig. 8:**
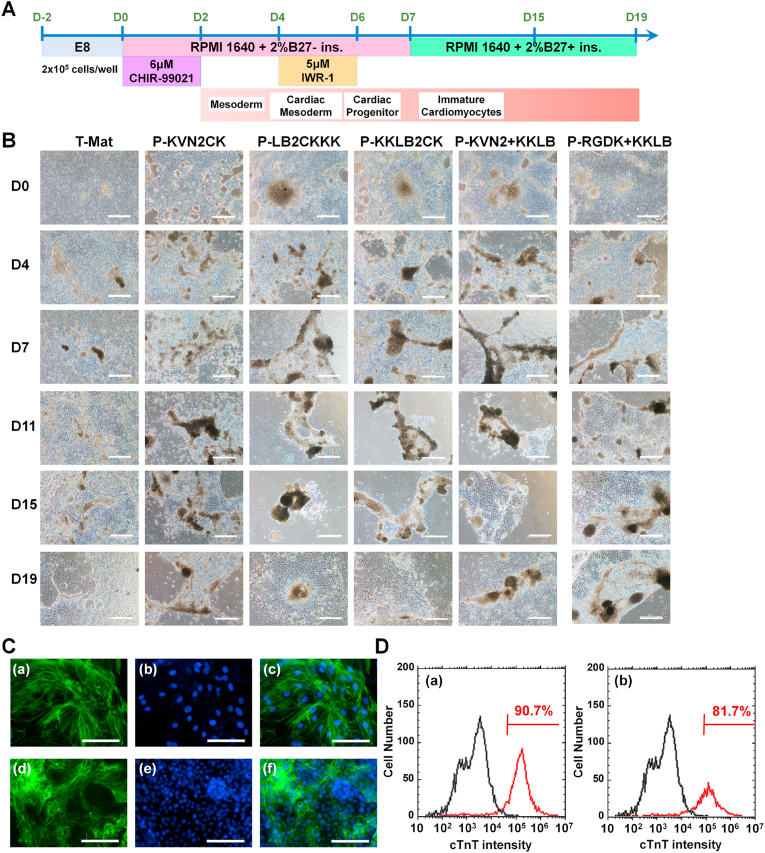
Cardiac differentiation of hiPSCs (HPS0077) after long-term (passage ten) culture on Matrigel-coated dishes and single or dual peptide-conjugated PVA hydrogels under xeno-free proliferation conditions. (A) Timeline of the cardiac induction protocol for hiPSCs used in this experiment. (B) Sequential morphological detection during cardiac induction of hiPSCs on Matrigel-coated surfaces and P-KVN2CK, P-LB2CKKK, P-KKLB2CK, P-VN2C + KKLB, and P-RGDK + KKLB hydrogels. Scale bar represents 500 μm. (C) Immunohistochemical staining of hiPSC-derived cardiomyocytes on P-VN2C + KKLB (a–c) and P-RGDK + KKLB (d–f) hydrogels after the long-term (ten passages) proliferation of hiPSCs on P-VN2C + KKLB hydrogels (a–c) and P-RGDK + KKLB hydrogels (d–f), respectively. Expression of cTnT (a, c, d, f, green) in hiPSC-derived cardiomyocytes, as determined with immunohistochemical staining, differentiated on P-VN2C + KKLB (a–c) and P-RGDK + KKLB (d–f) hydrogels on day 17. DAPI (b and e, blue) was used to stain nuclei. The photos in (c) and (f) were created by merging (a)–(b) and (d)–(e), respectively. The scale bar represents 100 μm. (D) Flow cytometry analysis of cardiac marker (cTnT) expression in hiPSC-derived cardiomyocytes differentiated after the long-term (ten passages) proliferation of hiPSCs on P-VN2C + KKLB hydrogels (a), and P-RGDK + KKLB hydrogels (b). (For interpretation of the references to color in this figure legend, the reader is referred to the Web version of this article.)

hiPSCs were differentiated into specific cell types of cardiomyocytes after 10 passages on peptide-conjugated PVA hydrogels. This experiment was conducted to confirm whether hiPSCs can differentiate into a specific type of cell, cardiomyocytes, after long-term culture on single or dual peptide-conjugated PVA hydrogel surfaces in.

xeno-free cultivation media (E8 media). After ten passages on peptide-conjugated PVA hydrogels, hiPSCs were differentiated into cardiomyocytes following a protocol using a GSK-3β inhibitor (CHIR99021) and Wnt inhibitor (IWR-1) (Fig. 8A) [41,42]. Fig. 8B illustrates the morphologies of hiPSCs during induction into cardiac cells at days 0, 4, 7, 11, 15 and 19. We proceeded hiPSC differentiation into cardiomyocytes, which were cultured on Matrigel-coated dishes (T-Mat) and single or dual peptide-conjugated PVA hydrogels. We did not include hiPSC differentiation into cardiomyocytes, which were cultured on rVN-coated dishes (T-rVN) as control experiments. This is because the cardiac differentiation of hiPSCs on T-rVN dishes showed very low successful efficiency as reported in a previous study [55]. The cells tended to aggregate with increasing differentiation time. We observed beating cells after 11 days of induction; Supplementary video 1 shows cardiac cells differentiated from hiPSCs cultured on P-RGDK + KKLB hydrogels, and Supplementary video 2 shows cardiomyocytes differentiated from hiPSCs cultured on P-VN2C + KKLB hydrogels. After 19 days of cardiac induction, using immunohistochemical methods, we studied the expression of the cardiac marker protein of cTnT in cardiac cells derived from hiPSCs (Fig. 8C) previously proliferated on P-RGDK + KKLB and P-VN2C + KKLB hydrogels for 10 passages. The hiPSC-derived cardiac cells extensively expressed the cardiac marker cTnT (Fig. 8D).

These results indicated that hiPSCs cultured on dual peptide-conjugated PVA hydrogels for ten passages in xeno-free proliferation medium maintained their potential to differentiate into specific cell lineages, such as cardiac cells, with excellent efficacy.

## 4 Conclusion

PVA hydrogels conjugated with dual integrin-targeting peptides (P-RGDK + KKLB and P-VN2C + KKLB hydrogels) were prepared for hPSC culture and differentiation. hPSCs proliferated on dual peptide-conjugated PVA hydrogels, which were prepared with 200 μg/mL peptide solution, for over ten passages and maintained their pluripotent conditions and potential to induce into cells derived from 3 germ layers; previously, peptide-conjugated biomaterials for hPSC culture were typically prepared with over 1000 μg/mL peptide solution [8,17,19,[26], [27], [28]]. In the peptides designed herein, dual chain peptides, a joint segment, and the addition of positive amino acids into the peptides enhanced the zeta potential, enabling hPSCs to proliferate on dual peptide-conjugated PVA hydrogels with excellent efficiency. However, ECM protein concentration, which is used for hPSC culture on ECM protein-coated dishes, is typically 5–10 μg/mL such as rVN-coated or LN521-coated dishes, whereas current ECM protein-derived peptide concentration on dual peptide-conjugated PVA hydrogels reported in this study is still 200 μg/mL. We need extra design of cell culture materials for the effective presentation of ECM protein-derived peptides for hPSCs on dual peptide-conjugated PVA hydrogels, which will be enable to use much less concentration (e.g., 10–100 μg/mL) of ECM protein-derived peptides in future. The genetic stability/karyotyping of the cells should be evaluated in future for application of the present dual peptide-conjugated PVA hydrogels in clinical application after long term culture of hPSCs.

## CRediT authorship contribution statement

**Tzu-Cheng Sung:** Visualization, Data curation. **Yen-Hung Chen:** Visualization, Data curation. **Ting Wang:** Data curation. **Liu Qian:** Data curation. **Wen-Hui Chao:** Data curation. **Jun Liu:** Data curation. **Jiandong Pang:** Supervision, Data curation. **Qing-Dong Ling:** Supervision, Data curation. **Henry Hsin-Chung Lee:** Supervision. **Akon Higuchi:** Writing – review & editing, Writing – original draft, Supervision, Resources, Methodology, Funding acquisition, Conceptualization.

## Declaration of competing interest

The authors declare the following financial interests/personal relationships which may be considered as potential competing interests:Akon Higuchi reports financial support was provided by 10.13039/501100012166National Key Research and Development Program of China. Akon Higuchi reports financial support was provided by 10.13039/501100001809National Natural Science Foundation of China. Tzu-Cheng Sung reports financial support was provided by 10.13039/501100007194Wenzhou Municipal Science and Technology Bureau. Akon Higuchi reports financial support was provided by 10.13039/501100007194Wenzhou Municipal Science and Technology Bureau. Akon Higuchi reports financial support was provided by 10.13039/501100011912Taipei Veterans General Hospital. Akon Higuchi reports financial support was provided by National Defense Medical Center. Henry Hsin-Chung Lee reports financial support was provided by 10.13039/501100002811Cathay General Hospital. Akon Higuchi reports financial support was provided by National Science and Technology Council. If there are other authors, they declare that they have no known competing financial interests or personal relationships that could have appeared to influence the work reported in this paper.

## Data Availability

Data will be made available on request.

## References

[1] Golchin A., Chatziparasidou A., Ranjbarvan P., Niknam Z., Ardeshirylajimi A. (2021). Embryonic stem cells in clinical trials: current overview of developments and challenges. Adv. Exp. Med. Biol..

[2] Deinsberger J., Reisinger D., Weber B. (2020). Global trends in clinical trials involving pluripotent stem cells: a systematic multi-database analysis. NPJ Regen Med.

[3] Higuchi A., Kumar S.S., Benelli G., Ling Q.D., Li H.F., Alarfaj A.A., Munusamy M.A., Sung T.C., Chang Y., Murugan K. (2019). Biomaterials used in stem cell therapy for spinal cord injury. Prog. Mater. Sci..

[4] Kobold S., Guhr A., Mah N., Bultjer N., Seltmann S., Seiler Wulczyn A.E.M., Stacey G., Jie H., Liu W., Loser P., Kurtz A. (2020). A manually curated database on clinical studies involving cell Products derived from human pluripotent stem cells. Stem Cell Rep..

[5] Higuchi A., Kumar S.S., Benelli G., Alarfaj A.A., Munusamy M.A., Umezawa A., Murugan K. (2017). Stem cell therapies for reversing vision loss. Trends Biotechnol..

[6] Fuchs S., van Helden R.W.J., Wiendels M., de Graaf M.N.S., Orlova V.V., Mummery C.L., van Meer B.J., Mayr T. (2022). On-chip analysis of glycolysis and mitochondrial respiration in human induced pluripotent stem cells. Mater Today Bio..

[7] Piantino M., Louis F., Shigemoto-Mogami Y., Kitamura K., Sato K., Yamaguchi T., Kawabata K., Yamamoto S., Iwasaki S., Hirabayashi H., Matsusaki M. (2022). Brain microvascular endothelial cells derived from human induced pluripotent stem cells as in vitro model for assessing blood-brain barrier transferrin receptor-mediated transcytosis. Mater. Today Bio..

[8] Wang T., Liu Q., Chang Y.T., Liu J., Yu T., Maitiruze K., Ban L.K., Sung T.C., Subbiah S.K., Renuka R.R., Jen S.H., Lee H.H., Higuchi A. (2023). Designed peptide-grafted hydrogels for human pluripotent stem cell culture and differentiation. J. Mater. Chem. B.

[9] Tian Z., Wang C.K., Lin F.L., Liu Q., Wang T., Sung T.C., Alarfaj A.A., Hirad A.H., Lee H.H.C., Wu G.J., Higuchi A. (2022). Effect of extracellular matrix proteins on the differentiation of human pluripotent stem cells into mesenchymal stem cells. J. Mater. Chem. B.

[10] Rodin S., Antonsson L., Niaudet C., Simonson O.E., Salmela E., Hansson E.M., Domogatskaya A., Xiao Z., Damdimopoulou P., Sheikhi M., Inzunza J., Nilsson A.S., Baker D., Kuiper R., Sun Y., Blennow E., Nordenskjold M., Grinnemo K.H., Kere J., Betsholtz C., Hovatta O., Tryggvason K. (2014). Clonal culturing of human embryonic stem cells on laminin-521/E-cadherin matrix in defined and xeno-free environment. Nat. Commun..

[11] Mesquita F.C.P., Leite E.S., Morrissey J., Freitas C., Coelho-Sampaio T., Hochman-Mendez C. (2022). Polymerized laminin-521: a feasible substrate for expanding induced pluripotent stem cells at a low protein concentration. Cells.

[12] Albalushi H., Kurek M., Karlsson L., Landreh L., Kjartansdottir K.R., Soder O., Hovatta O., Stukenborg J.B. (2018). Laminin 521 stabilizes the pluripotency expression pattern of human embryonic stem cells initially derived on feeder cells. Stem Cell. Int..

[13] Melkoumian Z., Weber J.L., Weber D.M., Fadeev A.G., Zhou Y., Dolley-Sonneville P., Yang J., Qiu L., Priest C.A., Shogbon C., Martin A.W., Nelson J., West P., Beltzer J.P., Pal S., Brandenberger R. (2010). Synthetic peptide-acrylate surfaces for long-term self-renewal and cardiomyocyte differentiation of human embryonic stem cells. Nat. Biotechnol..

[14] Kawase E., Nakatsuji N. (2023). Development of substrates for the culture of human pluripotent stem cells. Biomater. Sci..

[15] Jin S., Yao H., Weber J.L., Melkoumian Z.K., Ye K. (2012). A synthetic, xeno-free peptide surface for expansion and directed differentiation of human induced pluripotent stem cells. PLoS One.

[16] Kawase E. (2016). Efficient expansion of dissociated human pluripotent stem cells using a synthetic substrate. Methods Mol. Biol..

[17] Wang M., Deng Y., Zhou P., Luo Z., Li Q., Xie B., Zhang X., Chen T., Pei D., Tang Z., Wei S. (2015). In vitro culture and directed osteogenic differentiation of human pluripotent stem cells on peptides-decorated two-dimensional microenvironment. ACS Appl. Mater. Interfaces.

[18] Dolley-Sonneville P.J., Romeo L.E., Melkoumian Z.K. (2013). Synthetic surface for expansion of human mesenchymal stem cells in xeno-free, chemically defined culture conditions. PLoS One.

[19] Alarfaj A.A., Hirad A.H., Munusamy M.A., Kumar S.S., Higuchi A. (2022). Human embryonic stem cells cultured on hydrogels grafted with extracellular matrix protein-derived peptides with polyethylene glycol joint nanosegments. IET Nanobiotechnol..

[20] Zhou P., Yin B., Zhang R., Xu Z.R., Liu Y.Q., Yan Y.B., Zhang X.H., Zhang S.Q., Li Y.L., Liu H.X., Yuan Y.A., Wei S.C. (2018). Molecular basis for RGD-containing peptides supporting adhesion and self-renewal of human pluripotent stem cells on synthetic surface. Colloids Surf., B.

[21] Valamehr B., Tsutsui H., Ho C.M., Wu H. (2011). Developing defined culture systems for human pluripotent stem cells. Regen. Med..

[22] Park W.U., Yeon G.B., Yu M.S., Goo H.G., Hwang S.H., Na D., Kim D.S. (2021). A novel vitronectin peptide facilitates differentiation of oligodendrocytes from human pluripotent stem cells (synthetic ECM for oligodendrocyte differentiation). Biology.

[23] Jia J., Jeon E.J., Li M., Richards D.J., Lee S., Jung Y., Barrs R.W., Coyle R., Li X., Chou J.C., Yost M.J., Gerecht S., Cho S.W., Mei Y. (2020). Evolutionarily conserved sequence motif analysis guides development of chemically defined hydrogels for therapeutic vascularization. Sci. Adv..

[24] Jia J., Coyle R.C., Richards D.J., Berry C.L., Barrs R.W., Biggs J., Chou C.J., Trusk T.C., Mei Y. (2016). Development of peptide-functionalized synthetic hydrogel microarrays for stem cell and tissue engineering applications. Acta Biomater..

[25] Sung T.C., Wang T., Liu Q., Ling Q.D., Subbiah S.K., Renuka R.R., Hsu S.T., Umezawa A., Higuchi A. (2023). Cell-binding peptides on the material surface guide stem cell fate of adhesion, proliferation and differentiation. J. Mater. Chem. B.

[26] Chen Y.M., Chen L.H., Li M.P., Li H.F., Higuchi A., Kumar S.S., Ling Q.D., Alarfaj A.A., Munusamy M.A., Chang Y., Benelli G., Murugan K., Umezawa A. (2017). Xeno-free culture of human pluripotent stem cells on oligopeptide-grafted hydrogels with various molecular designs. Sci. Rep..

[27] Higuchi A., Kao S.H., Ling Q.D., Chen Y.M., Li H.F., Alarfaj A.A., Munusamy M.A., Murugan K., Chang S.C., Lee H.C., Hsu S.T., Kumar S.S., Umezawa A. (2015). Long-term xeno-free culture of human pluripotent stem cells on hydrogels with optimal elasticity. Sci. Rep..

[28] Sung T.C., Lu M.W., Tian Z., Lee H.H., Pan J., Ling Q.D., Higuchi A. (2021). Poly(vinyl alcohol-co-itaconic acid) hydrogels grafted with several designed peptides for human pluripotent stem cell culture and differentiation into cardiomyocytes. J. Mater. Chem. B.

[29] Abdal Dayem A., Lee S.B., Lim K.M., Kim A., Shin H.J., Vellingiri B., Kim Y.B., Cho S.G. (2023). Bioactive peptides for boosting stem cell culture platform: methods and applications. Biomed. Pharmacother..

[30] Liu Y., Chakraborty S., Direksilp C., Scheiger J.M., Popova A.A., Levkin P.A. (2021). Miniaturized droplet microarray platform enables maintenance of human induced pluripotent stem cell pluripotency. Mater Today Bio..

[31] Sun H., Chan Y., Li X., Xu R., Zhang Z., Hu X., Wu F., Deng F., Yu X. (2022). Multi-omics analysis of oral bacterial biofilm on titanium oxide nanostructure modified implant surface: in vivo sequencing-based pilot study in beagle dogs. Mater Today Bio..

[32] Peng I.C., Yeh C.C., Lu Y.T., Muduli S., Ling Q.D., Alarfaj A.A., Munusamy M.A., Kumar S.S., Murugan K., Lee H.C., Chang Y., Higuchi A. (2016). Continuous harvest of stem cells via partial detachment from thermoresponsive nanobrush surfaces. Biomaterials.

[33] Sung T.C., Yang J.S., Yeh C.C., Liu Y.C., Jiang Y.P., Lu M.W., Ling Q.D., Kumar S.S., Chang Y., Umezawa A., Chen H., Higuchi A. (2019). The design of a thermoresponsive surface for the continuous culture of human pluripotent stem cells. Biomaterials.

[34] Tian Z., Wang C.K., Lin F.L., Liu Q., Wang T., Sung T.C., Alarfaj A.A., Hirad A.H., Lee H.H., Wu G.J., Higuchi A. (2022). Effect of extracellular matrix proteins on the differentiation of human pluripotent stem cells into mesenchymal stem cells. J. Mater. Chem. B.

[35] Liu Y.C., Ban L.K., Lee H.H.C., Lee H.T., Chang Y.T., Lin Y.T., Su H.Y., Hsu S.T., Higuchi A. (2021). Laminin-511 and recombinant vitronectin supplementation enables human pluripotent stem cell culture and differentiation on conventional tissue culture polystyrene surfaces in xeno-free conditions. J. Mater. Chem. B.

[36] Higuchi A., Hirad A.H., Kumar S.S., Munusamy M.A., Alarfaj A.A. (2020). Thermoresponsive surfaces designed for the proliferation and differentiation of human pluripotent stem cells. Acta Biomater..

[37] Sung T.C., Li H.F., Higuchi A., Kumar S.S., Ling Q.D., Wu Y.W., Burnouf T., Nasu M., Umezawa A., Lee K.F., Wang H.C., Chang Y., Hsu S.T. (2020). Effect of cell culture biomaterials for completely xeno-free generation of human induced pluripotent stem cells. Biomaterials.

[38] Esser T.U., Trossmann V.T., Lentz S., Engel F.B., Scheibel T. (2021). Designing of spider silk proteins for human induced pluripotent stem cell-based cardiac tissue engineering. Mater Today Bio..

[39] Park J., Lee N.G., Oh M., Song J., Kim W., Kwon M.G., Kim S.G., Han B.S., Bae K.H., Lee D.G., Lee S.H., Park J.G., Kim J.H., Lee J., Min J.K. (2020). Selective elimination of human pluripotent stem cells by Anti-Dsg2 antibody-doxorubicin conjugates. Biomaterials.

[40] Kim K.T., Park J.C., Jang H.K., Lee H., Park S., Kim J., Kwon O.S., Go Y.H., Jin Y., Kim W., Lee J., Bae S., Cha H.J. (2020). Safe scarless cassette-free selection of genome-edited human pluripotent stem cells using temporary drug resistance. Biomaterials.

[41] Sharma A., Li G., Rajarajan K., Hamaguchi R., Burridge P.W., Wu S.M. (2015). Derivation of highly purified cardiomyocytes from human induced pluripotent stem cells using small molecule-modulated differentiation and subsequent glucose starvation. J. Vis. Exp..

[42] Sung T.C., Su H.C., Ling Q.D., Kumar S.S., Chang Y., Hsu S.T., Higuchi A. (2020). Efficient differentiation of human pluripotent stem cells into cardiomyocytes on cell sorting thermoresponsive surface. Biomaterials.

[43] Gao T., Zhao X., Hao J., Tian Y., Ma H., Liu W., An B., Sun F., Liu S., Guo B., Niu S., Li Z., Wang C., Wang Y., Feng G., Wang L., Li W., Wu J., Guo M., Zhou Q., Gu Q. (2023). A scalable culture system incorporating microcarrier for specialised mesenchymal stem cells from human embryonic stem cells. Mater Today Bio.

[44] Burridge P.W., Matsa E., Shukla P., Lin Z.C., Churko J.M., Ebert A.D., Lan F., Diecke S., Huber B., Mordwinkin N.M., Plews J.R., Abilez O.J., Cui B., Gold J.D., Wu J.C. (2014). Chemically defined generation of human cardiomyocytes. Nat. Methods.

[45] Fowler E.W., Ravikrishnan A., Witt R.L., Pradhan-Bhatt S., Jia X. (2021). RGDSP-decorated hyaluronate hydrogels facilitate rapid 3D expansion of amylase-expressing salivary gland progenitor cells. ACS Biomater. Sci. Eng..

[46] Arya N., Forget A., Sarem M., Shastri V.P. (2019). RGDSP functionalized carboxylated agarose as extrudable carriers for chondrocyte delivery. Mater. Sci. Eng., C.

[47] Schuler M., Owen G.R., Hamilton D.W., de Wild M., Textor M., Brunette D.M., Tosatti S.G. (2006). Biomimetic modification of titanium dental implant model surfaces using the RGDSP-peptide sequence: a cell morphology study. Biomaterials.

[48] Fowler E.W., van Venrooy E.J., Witt R.L., Jia X. (2022). A TGFbetaR inhibitor represses keratin-7 expression in 3D cultures of human salivary gland progenitor cells. Sci. Rep..

[49] Li X.F., Jia J., Mei Y., Latour R.A. (2017). Molecular modeling to predict peptide accessibility for peptide-functionalized hydrogels. Biointerphases.

[50] Wieduwild R., Howarth M. (2018). Assembling and decorating hyaluronan hydrogels with twin protein superglues to mimic cell-cell interactions. Biomaterials.

[51] Xu Y., Gaillez M.P., Zheng K., Voigt D., Cui M., Kurth T., Xiao L., Rothe R., Hauser S., Lee P.W., Wieduwild R., Lin W., Bornhauser M., Pietzsch J., Boccaccini A.R., Zhang Y. (2022). A self-assembled matrix system for cell-bioengineering applications in different dimensions, scales, and geometries. Small.

[52] Binner M., Bray L.J., Friedrichs J., Freudenberg U., Tsurkan M.V., Werner C. (2017). Cell-instructive starPEG-heparin-collagen composite matrices. Acta Biomater..

[53] Ravikrishnan A., Fowler E.W., Stuffer A.J., Jia X. (2021). Hydrogel-supported, engineered model of vocal fold epithelium. ACS Biomater. Sci. Eng..

[54] Tondera C., Wieduwild R., Röder E., Werner C., Zhang Y.X., Pietzsch J. (2017). In vivo examination of an injectable hydrogel system crosslinked by peptide-oligosaccharide interaction in immunocompetent nude mice. Adv. Funct. Mater..

[55] Sung T.C., Liu C.H., Huang W.L., Lee Y.C., Kumar S.S., Chang Y., Ling Q.D., Hsu S.T., Higuchi A. (2019). Efficient differentiation of human ES and iPS cells into cardiomyocytes on biomaterials under xeno-free conditions. Biomater Sci-Uk.

