# Supplementary material for: Design of dual peptide-conjugated hydrogels for proliferation and differentiation of human pluripotent stem cells
Source: Mater Today Bio. 2024 Jan 23;25:100969. doi: 10.1016/j.mtbio.2024.100969 (PMC10839443; doi:10.1016/j.mtbio.2024.100969)
Supplement: Multimedia component 1 [file mmc1.pdf]

Design of dual peptide-conjugated hydrogels for proliferation and differentiation of human pluripotent stem cells

Tzu-Cheng Sung[a,1], Yen-Hung Chen[b,1], Ting Wang[a,1], Liu Qian[a], Wen-Hui Chao[b], Jun Liu[a], Jiandong Pang[a], Qing-Dong Ling[c], Henry Hsin-Chung Lee[d,e,**], Akon Higuchi[a,b,f,*]

[a]School of Ophthalmology and Optometry, Eye Hospital, Wenzhou Medical University, No. 270, Xueyuan Road, Wenzhou, Zhejiang, 325027, China

[b]Department of Chemical and Materials Engineering, National Central University, No. 300, Jhongda RD., Jhongli, Taoyuan 32001, Taiwan

[c]Cathay Medical Research Institute, Cathay General Hospital, No. 32, Ln 160, Jian-Cheng Road, Hsi-Chi City, Taipei 221, Taiwan

[d]Department of Surgery, Hsinchu Cathay General Hospital, No. 678, Sec 2, Zhonghua Rd., Hsinchu, 30060, Taiwan

[e]Graduate Institute of Translational and Interdisciplinary Medicine, National Central University, No. 300, Jhongda Rd., Jhongli, Taoyuan, 32001, Taiwan

[f]R&D Center for Membrane Technology, Chung Yuan Christian University, Chungli, Taoyuan 320, Taiwan

* Corresponding author. Department of Chemical and Materials Engineering, National Central University, No. 300, Jhongda RD., Jhongli, Taoyuan 32001, Taiwan & School of Ophthalmology and Optometry, Eye Hospital, Wenzhou Medical University, No. 270, Xueyuan Road, Wenzhou, Zhejiang, 325027, China
Tel.: +86 577-88068822; fax: +86 086-577-88832083.
E-mail address: higuchi@ncu.edu.tw; higuchi@wmu.edu.cn (A. Higuchi)

** Corresponding author. Department of Surgery, Hsinchu Cathay General Hospital, No. 678, Sec 2, Zhonghua Rd., Hsinchu, 30060, Taiwan
E-mail address: hsinchuoff@cgh.org.tw (H.H.C. Lee)

[1] These authors contributed equally.

Supplementary Information

Supplementary Table 1 Materials used in this study.

Materials	Abbreviation	Catalog No.	Company
		**ECM**	
Matrigel	MAT	356230	Corning (Corning, NY, USA)
Recombinant vitronectin	rVN	A14700	Thermo Fisher Scientific Inc. (Waltham, MA, USA)
	FN		
	Coll I		
		**Cell culture dishes**	
6-well polystyrene plate	TCPS	#353046	Corning (Corning, NY, USA)
		**Chemicals**	
N-(3-dimethylaminopropyl)-N'-ethylcarbodiimide hydrochloride	EDC	E7750	Sigma-Aldrich (St. Louis, MO, USA)
N-Hydroxysuccinimide	NHS	56480	Sigma-Aldrich (St. Louis, MO, USA)
Dispase II	Dispase	D4693-1G	Sigma-Aldrich (St. Louis, MO, USA)
Trypsin-EDTA (0.25%)	Trypsin-EDTA	25200072	Thermo Fisher Scientific Inc. (Waltham, MA, USA)
B-27™ Supplement, minus insulin	B27-	A1895601	Thermo Fisher Scientific Inc. (Waltham, MA, USA)
B-27™ Supplement	B27	17504044	Thermo Fisher Scientific Inc. (Waltham, MA, USA)
CHIR99021	CHIR99021	SML1046	Sigma-Aldrich (St. Louis, MO, USA)
IWR-1	IWR-1	I0161	Sigma-Aldrich (St. Louis, MO, USA)
		**Cell culture medium and component**	
Essential 8 medium	Essential 8	A1517001	Thermo Fisher Scientific Inc. (Waltham, MA, USA)
Essential 6 medium	Essential 6	A1516401	Thermo Fisher Scientific Inc. (Waltham, MA, USA)
DMEM/F12 medium	DMEM/F12 medium	11330-057	Thermo Fisher Scientific Inc. (Waltham, MA, USA)
RPMI1640	RPMI1640	12633012	Thermo Fisher Scientific Inc. (Waltham, MA, USA)
		**Antibodies**	
Mouse Anti-GFAP	Anti-GFAP antibody	MA5-15086	Thermo Fisher Scientific Inc. (Waltham, MA, USA)
Mouse Anti-AFP	Anti-AFP antibody	PA5-21004	Thermo Fisher Scientific Inc. (Waltham, MA, USA)
Rabbit Anti-SMA	Anti-SMA antibody	PA5-19465	Thermo Fisher Scientific Inc. (Waltham, MA, USA)
Alexa Fluor® 488 Goat Anti-Rabbit IgG Antibody	488 Anti-Rabbit IgG Antibody	A11008	Thermo Fisher Scientific Inc. (Waltham, MA, USA)
Alexa Fluor® 488 Goat Anti-mouse IgG Antibody	488 Anti-Mouse IgG Antibody	A11001	Thermo Fisher Scientific Inc. (Waltham, MA, USA)
Alexa Fluor® 555 Goat Anti-Rabbit IgG Antibody	594 Donkey Anti-Rabbit IgG Antibody	A21428	Thermo Fisher Scientific Inc. (Waltham, MA, USA)
Alexa Fluor® 594 Donkey Anti-Mouse IgG (H+L) Antibody	594 Donkey Anti-Mouse IgG (H+L) Antibody	A21203	Thermo Fisher Scientific Inc. (Waltham, MA, USA)
Anti-cTnT antibody	Anti-cTnT antibody	Ab209813	Abcam (Cambridge, UK)
Rabbit IgG isotype	Rabbit IgG isotype	10500C	Thermo Fisher Scientific Inc. (Waltham, MA, USA)
Anti-Rabbit IgG (FITC)	Anti-Rabbit IgG	Ab6717	Abcam (Cambridge, UK)

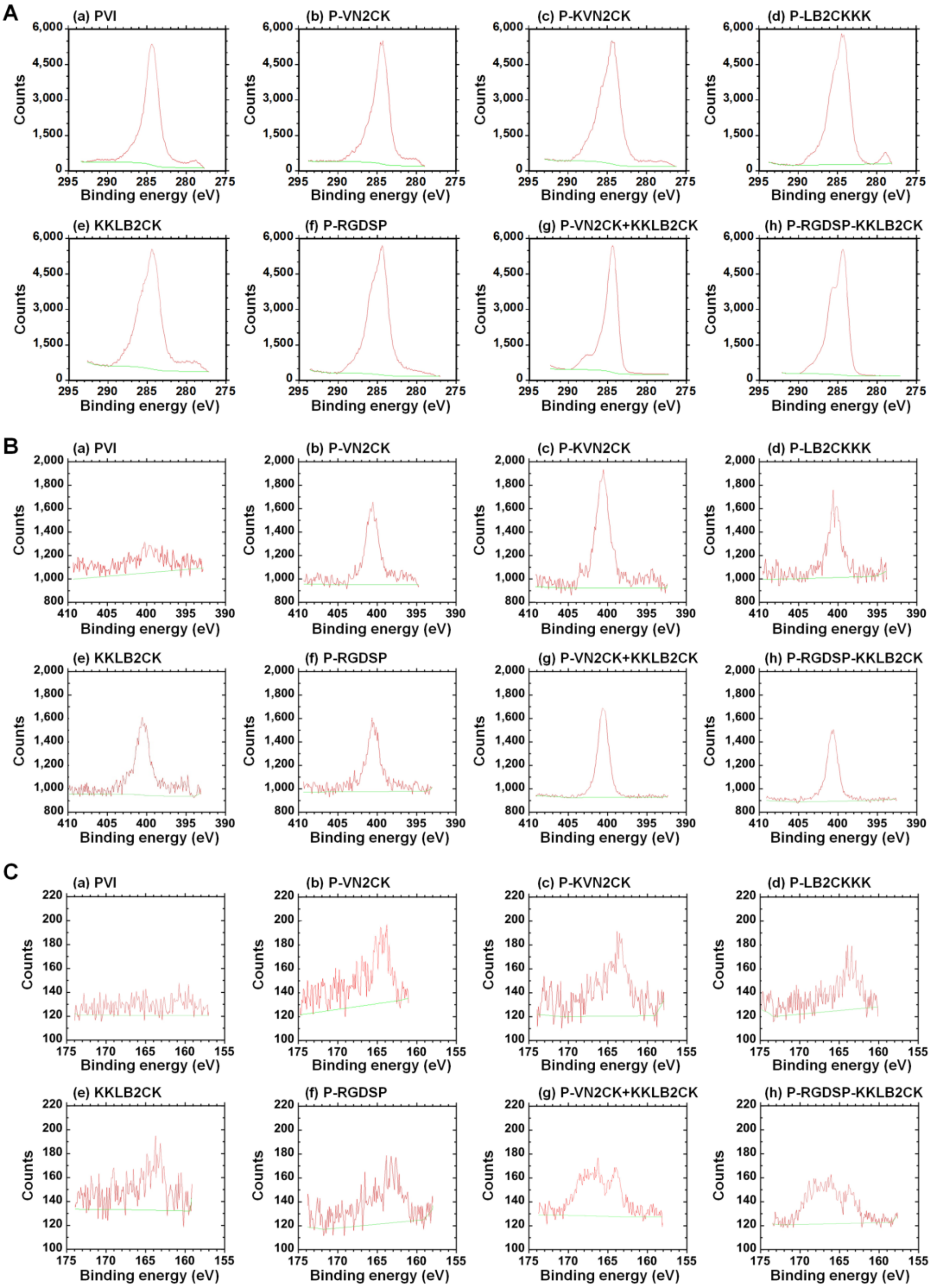

Supplementary Fig. 1 Surface analysis of peptide-conjugated PVA hydrogels by XPS. (A, B, C) High-resolution spectra of the C 1s (A), N 1s (B), and S 2p (C) peaks for the surfaces of the PVA (a), P-VN2CK (b), P-KVN2CK (c), P-LB2CKKK (d), P-KKLB2CK (e), P-RGDSP (f), P-VN2C+KKLB (g), and P-RGDS+KKLB (h) hydrogels.

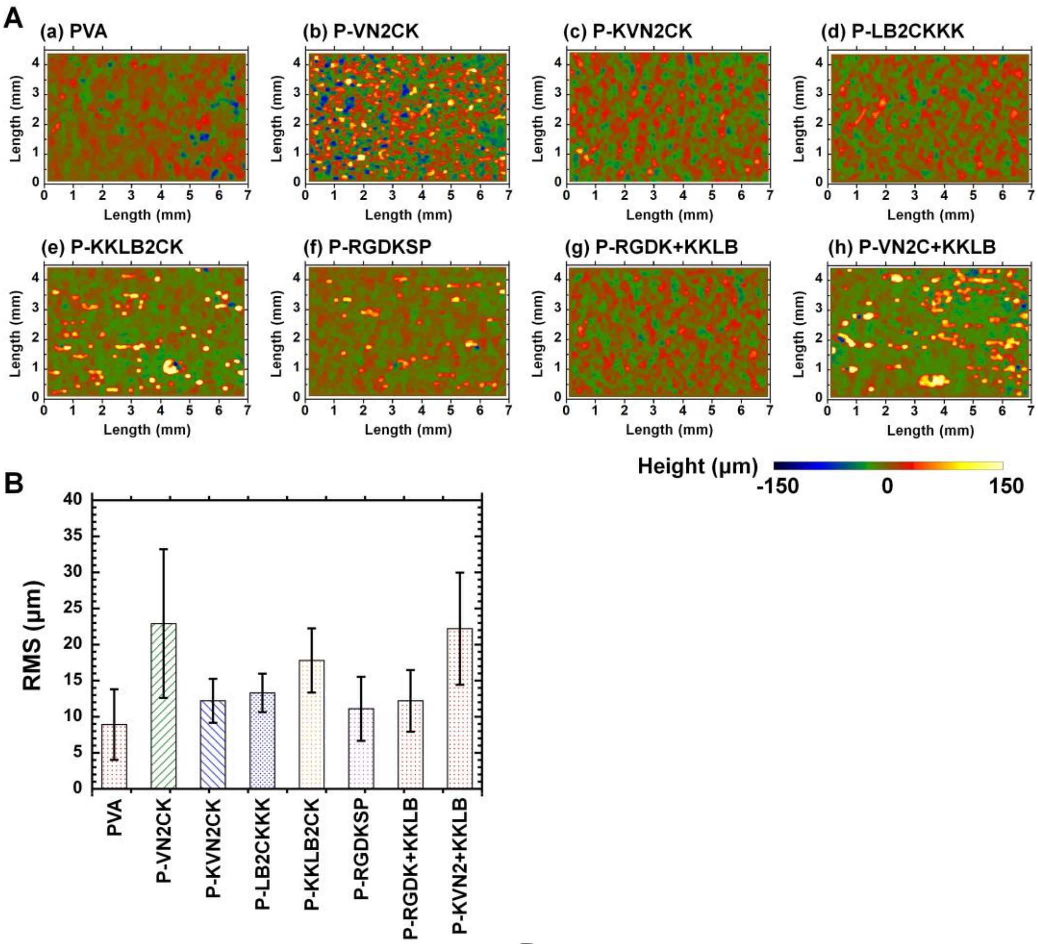

Supplementary Fig. 2 Physical characterization of peptide-conjugated PVA hydrogels. (A) Surface roughness of PVA (PV) hydrogels (a), P-VN2CK hydrogels (b), P-KVN2CK hydrogels (c), P-LB2CKKK (d), P-KKLB2CK (e), P-RGDKSP (f), P-RGDK+KKLB (g), and P-VN2C+KKLB (h), as evaluated utilizing PRIMOS CR45. (B) RMS roughness of the peptide-conjugated PVA hydrogel surfaces, as evaluated utilizing PRIMOS CR45.

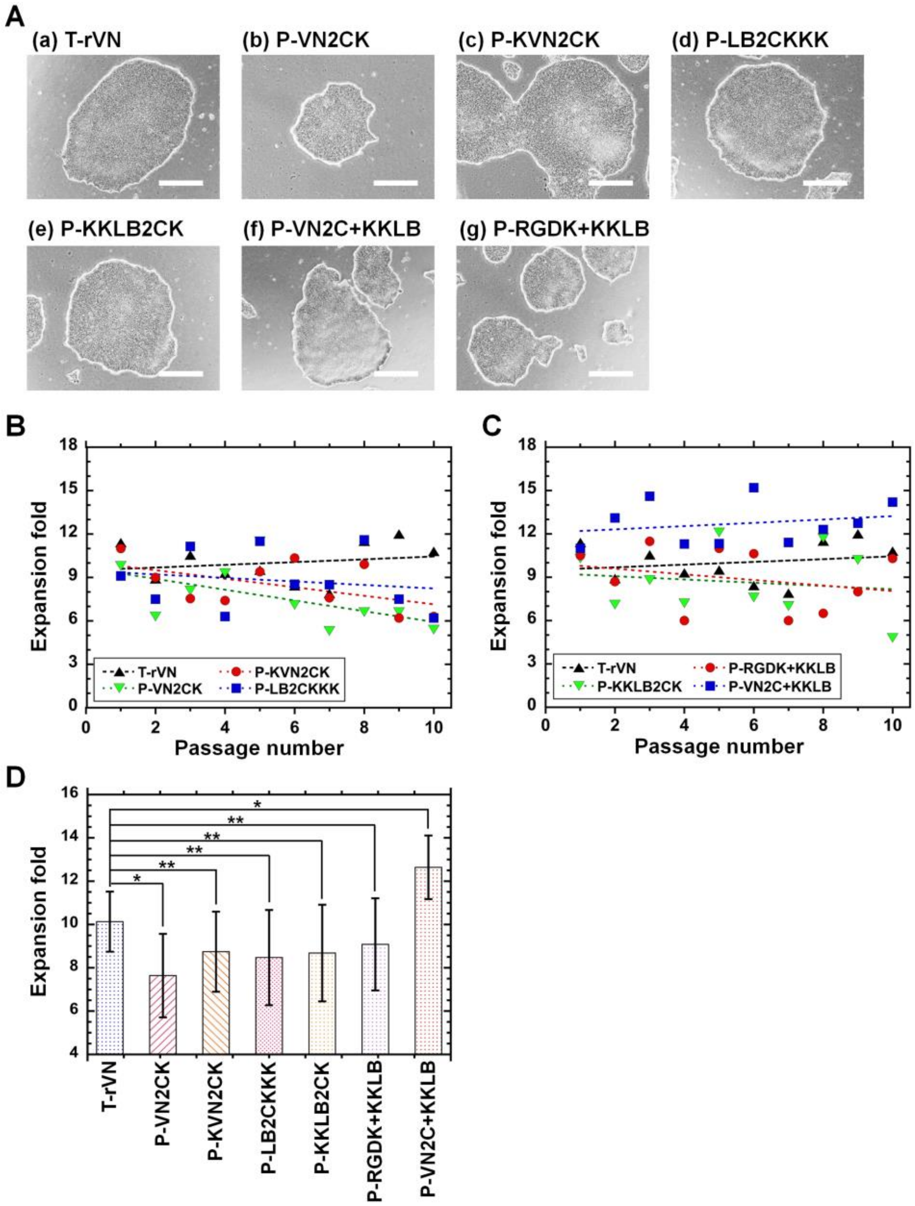

Supplementary Fig. 3 Long-term proliferation of hESCs (H9) on peptide-conjugated PVA hydrogels under xeno-free proliferation conditions. (A) Morphologies of hESCs on rVN-coated dishes (a), P-VN2CK hydrogels (b), P-KVN2CK hydrogels (c), P-LB2CKKK hydrogels (d), P-KKLB2CK (e), P-VN2C+KKLB hydrogels (f), and P-RGDK+KKLB hydrogels (g) at passage ten. The scale bar represents 500 μm. (B) Dependence of fold expansion of hESCs on passage on rVN-coated dishes (closed black triangle), P-VN2CK hydrogels (closed reverse green triangle), P-KVN2CK hydrogels (closed red circle), and P-LB2CKKK (closed blue square). (C) Dependence of fold expansion of hESCs on passage on rVN-coated dishes (closed black triangle), P-KKLB2CK hydrogels (closed reverse green trianle), P-RGDK+KKLB hydrogels (closed red circle), and P-VN2C+KKLB hydrogels (closed blue square). (D) Average fold expansion of hiPSCs on rVN-coated dishes and single or dual peptide-conjugated PVA hydrogels for 10 passages. * $p < 0.05$. ** $p > 0.05$.

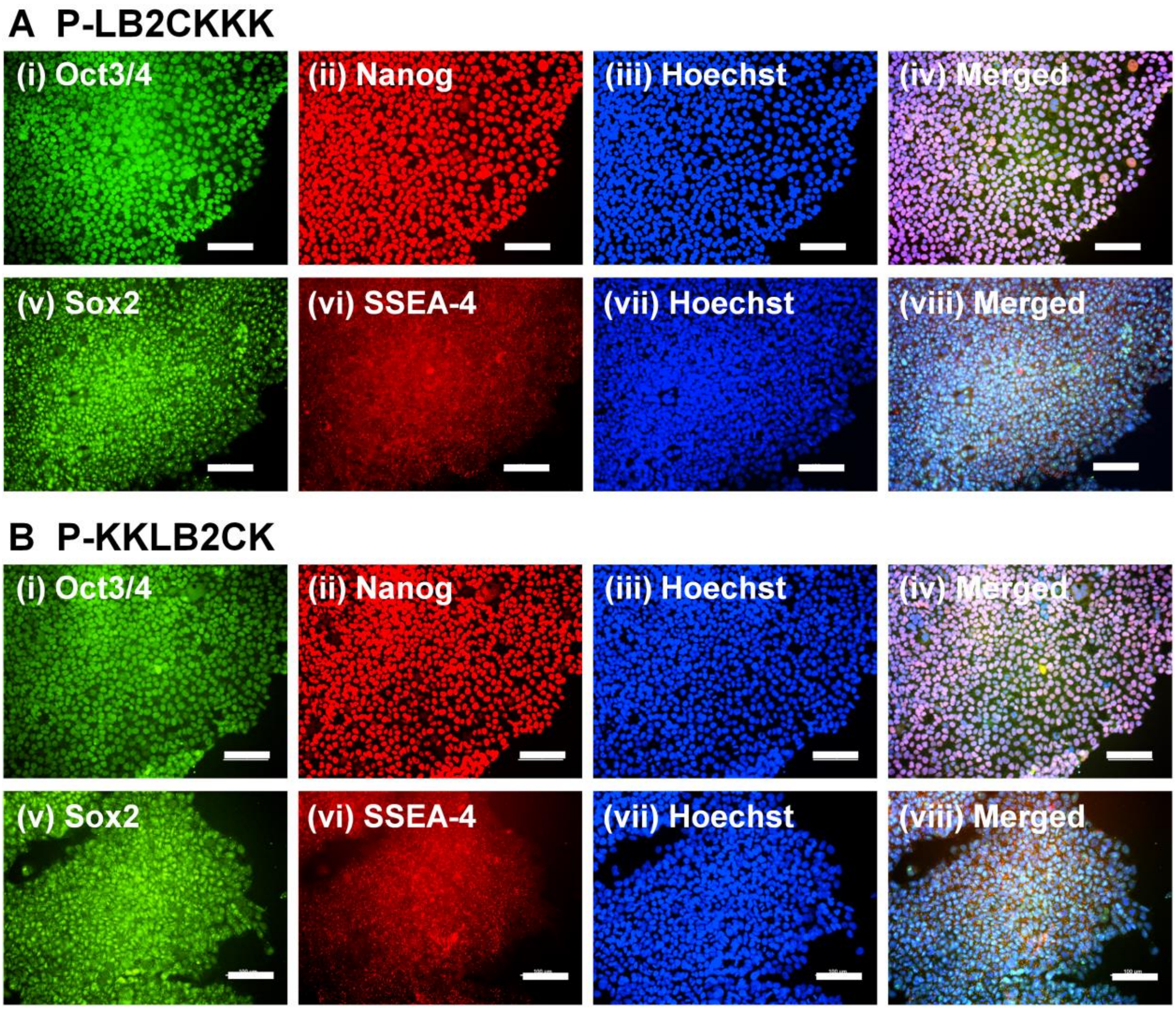

Supplementary Fig. 4. Analysis of the pluripotency of hiPSCs after long-term (ten passages) proliferation on peptide-conjugated PVA hydrogels under xeno-free proliferation conditions. (A, B) Expression of the pluripotent proteins Oct3/4 (i, green), Nanog (ii, red), Sox2 (v, green), and SSEA-4 (vi, red) in hiPSCs, as determined with immunostaining and nuclear staining (Hoechst 33342) (blue, iii, vii), after the long-term (ten passages) proliferation of hiPSCs on P-LB2CKKK (A) and P-KKLB2CK (B) hydrogels. The images in (iv) and (viii) were created by merging (i)–(iii) and (v)–(vii), respectively. The scale bar represents 100 µm.

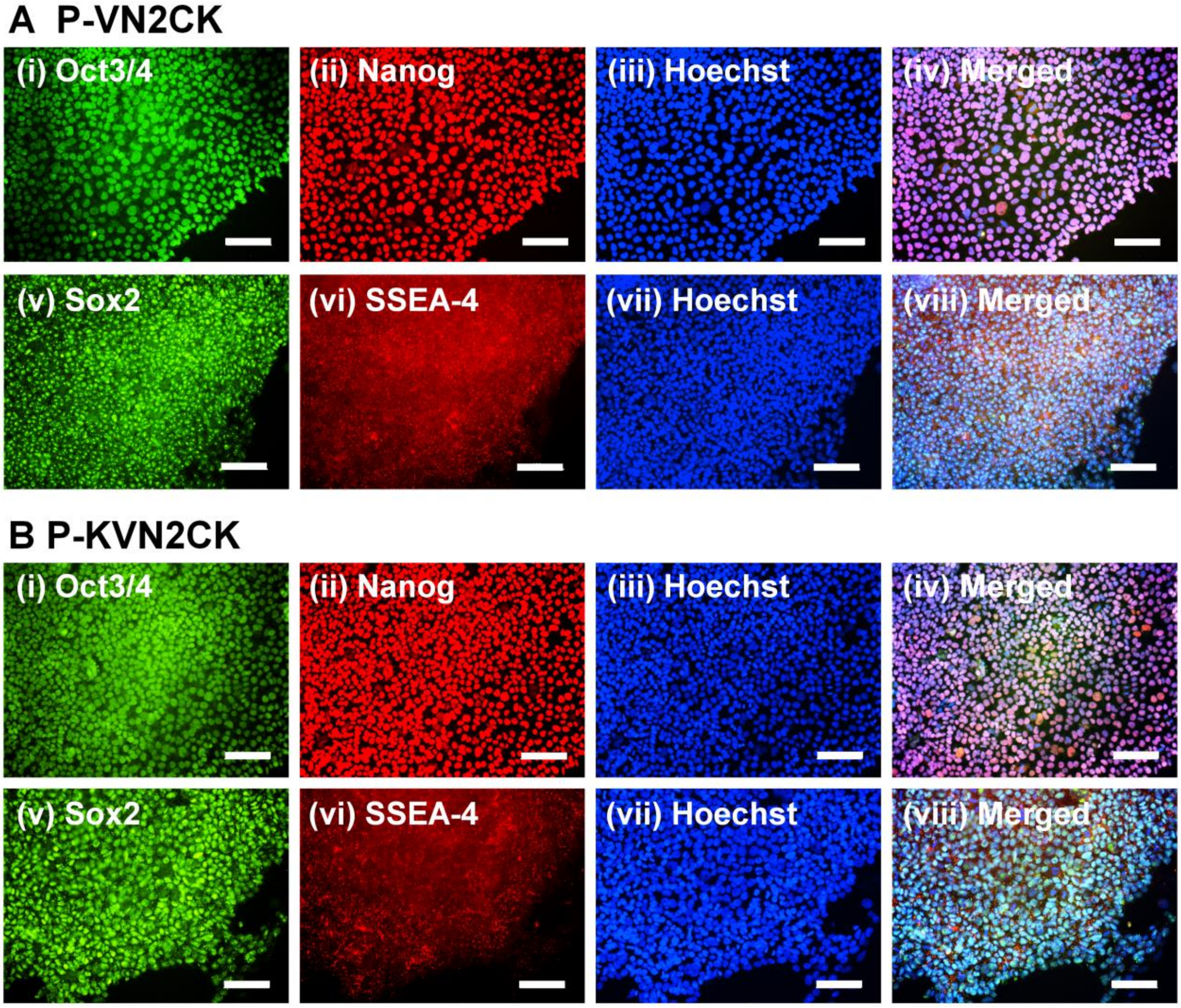

Supplementary Fig. 5. Analysis of the pluripotency of hiPSCs after long-term (ten passages) proliferation on peptide-conjugated PVA hydrogels under xeno-free proliferation conditions. (A, B) Expression of the pluripotency proteins Oct3/4 (i, green), Nanog (ii, red), Sox2 (v, green), and SSEA-4 (vi, red) in hiPSCs, as determined with immunostaining and nuclear staining (Hoechst 33342) (blue, iii, vii) after the long-term (ten passages) proliferation of hiPSCs on P-VN2CK (A) and P-KVN2CK (B) hydrogels. The images in (iv) and (viii) were created by merging (i)–(iii) and (v)–(vii), respectively. The scale bar represents 100 μm.

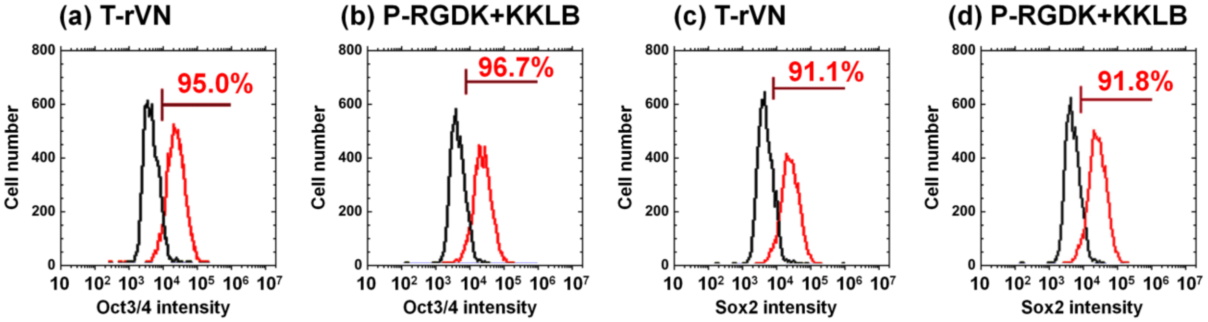

Supplementary Fig. 6. Flow cytometry analysis of pluripotent marker, Oct3/4 (a, b) and Sox2 (c, d), expression in hiPSCs after long-term (ten passages) proliferation on rVN-coated dishes (a, c) and P-VN2C+KKLB hydrogels (b, d).

Supplementary Video 1. hiPSC-derived cardiac cells prepared on P-RGDK+KKLB hydrogels.

Supplementary Video 2. hiPSC-derived cardiac cells prepared on P-VN2C+KKLB hydrogels.
